# Supplementary material for: Bayesian reweighting of biomolecular structural ensembles using heterogeneous cryo-EM maps with the cryoENsemble method
Source: Sci Rep. 2024 Aug 5;14:18149. doi: 10.1038/s41598-024-68468-7 (PMC11300795; doi:10.1038/s41598-024-68468-7)
Supplement: Supplementary file 1 — Supplementary Figures. [file 41598_2024_68468_MOESM1_ESM.docx]

**SUPPLEMENTARY INFORMATION**

**Bayesian reweighting of biomolecular structural ensembles using heterogeneous cryo-EM maps with the cryoENsemble method**

Tomasz Włodarski^1,2*^, Julian O. Streit^1^, Alkistis Mitropoulou^1^, Lisa D. Cabrita^1^, Michele Vendruscolo^4^ and John Christodoulou^1,3^

*^1^Institute of Structural and Molecular Biology, University College London, UK*

*^2^Institute of Biochemistry and Biophysics, Polish Academy of Sciences, Warsaw, Poland*

*^3^Birkbeck College, University of London, UK*

*^4^Centre for Misfolding Diseases, Yusuf Hamied Department of Chemistry,*

*University of Cambridge, Cambridge, UK*


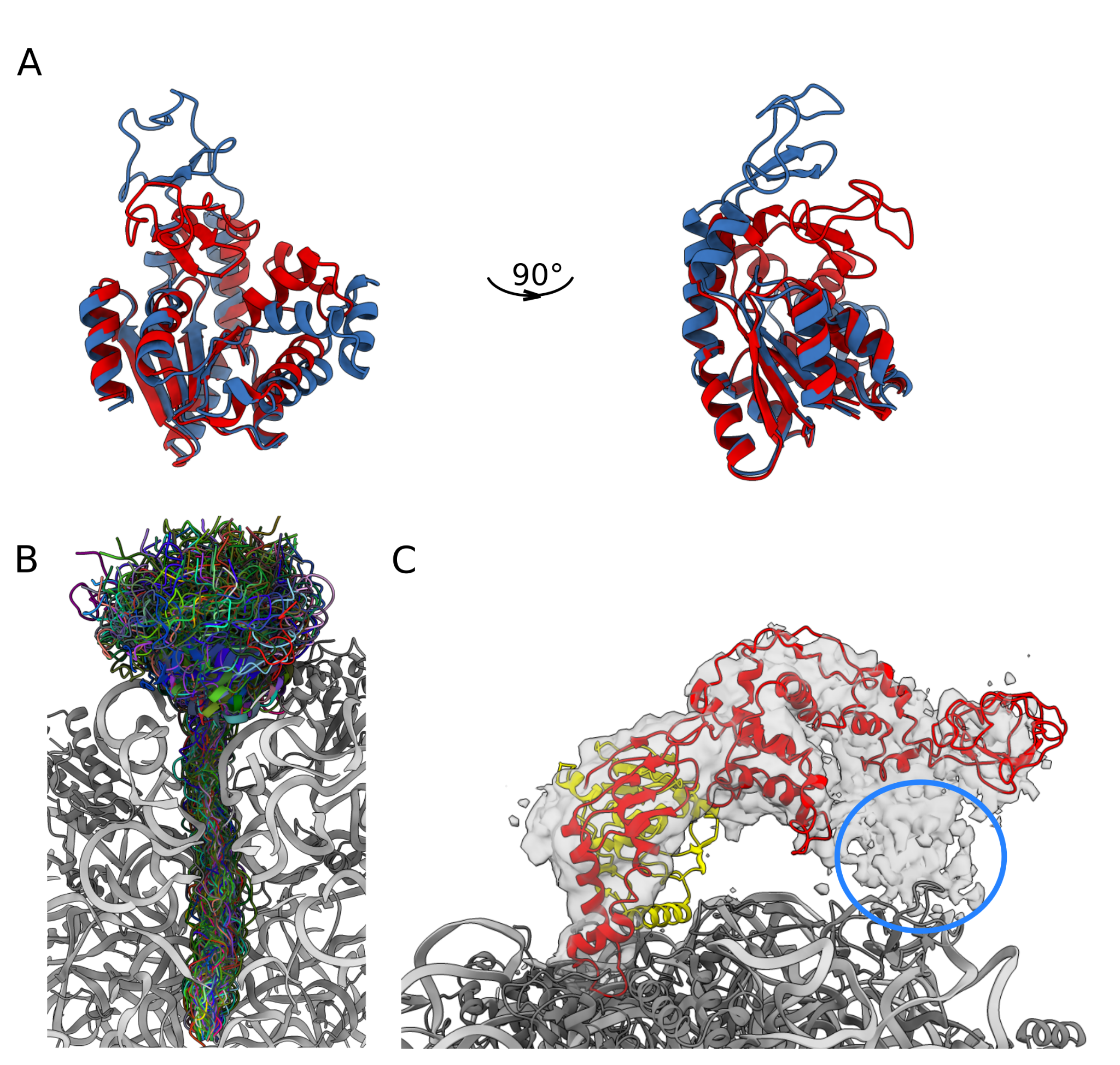


**Supplementary Figure 1.** Molecular systems used for the development and testing of the cryoENsemble method. **(A)** X-ray structures of the *E. coli* adenylate kinase (ADK) in the open (PDB ID: 4AKE[^1^](https://sciwheel.com/work/citation?ids=322311&pre=&suf=&sa=0), shown in blue) and closed (PDB ID: 1AKE[^2^](https://sciwheel.com/work/citation?ids=1560841&pre=&suf=&sa=0), shown in red) states. **(B)** Structural ensemble of FLN5-6 ribosome nascent chain complex (FLN5-6 RNC) consisting of 100 structures, randomly selected from the all-atom structure-based MD simulation[^3^](https://sciwheel.com/work/citation?ids=13359631&pre=&suf=&sa=0) that are used in the reweighting process. Each structure is depicted in a different colour and combines the N-terminal folded FLN5 domain followed by 31 amino acid linker consisting of the subsequent FLN6 domain and a SecM stalling sequence covalently bound to the tRNA at the peptidyl transferase centre. A cross-section of the 70S ribosome is shown for clarity (in grey ribosomal proteins and in silver rRNA). **(C)** Trigger factor (in red) and peptide deformylase (in yellow) bound to the 70S ribosome (ribosomal proteins in grey, rRNA in silver) (from PDB ID: 7D80[^4^](https://sciwheel.com/work/citation?ids=10778241&pre=&suf=&sa=0) with cryo-EM density corresponding to TF bound states. The region of incompletely characterised density is depicted by a blue circle (from EMDB: 30611[^4^](https://sciwheel.com/work/citation?ids=10778241&pre=&suf=&sa=0)).

**
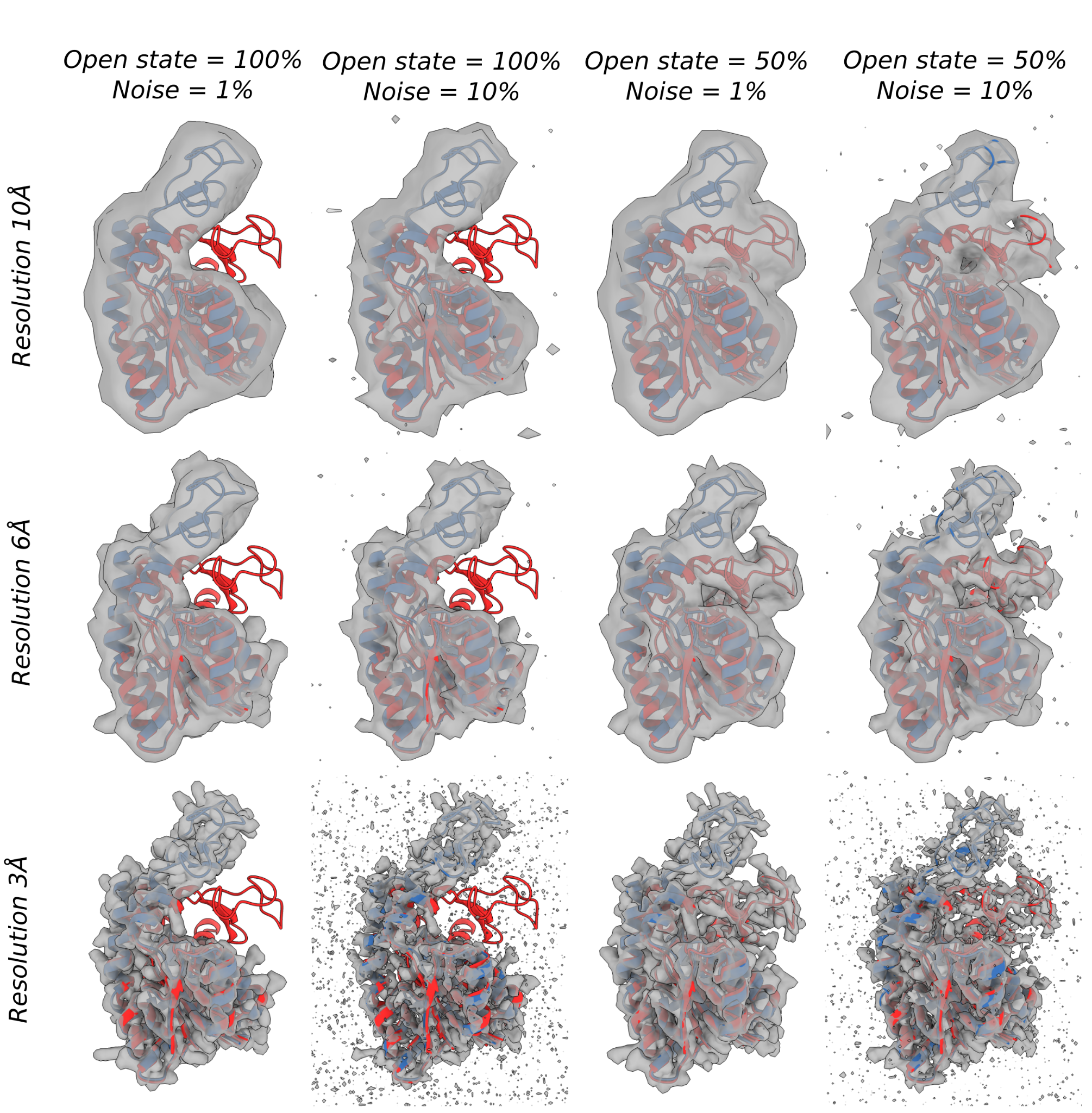
**

**Supplementary Figure 2.** Representative structures and density maps of ADK. ADK X-ray structures in the open (shown in blue) and closed (shown in red) states, along with their generated density maps. The reference density maps were generated based on the varied populations of the open and closed states, different map resolutions, and noise levels. All density maps are depicted at a threshold level equal to three times the standard deviation of the noise distribution.

**
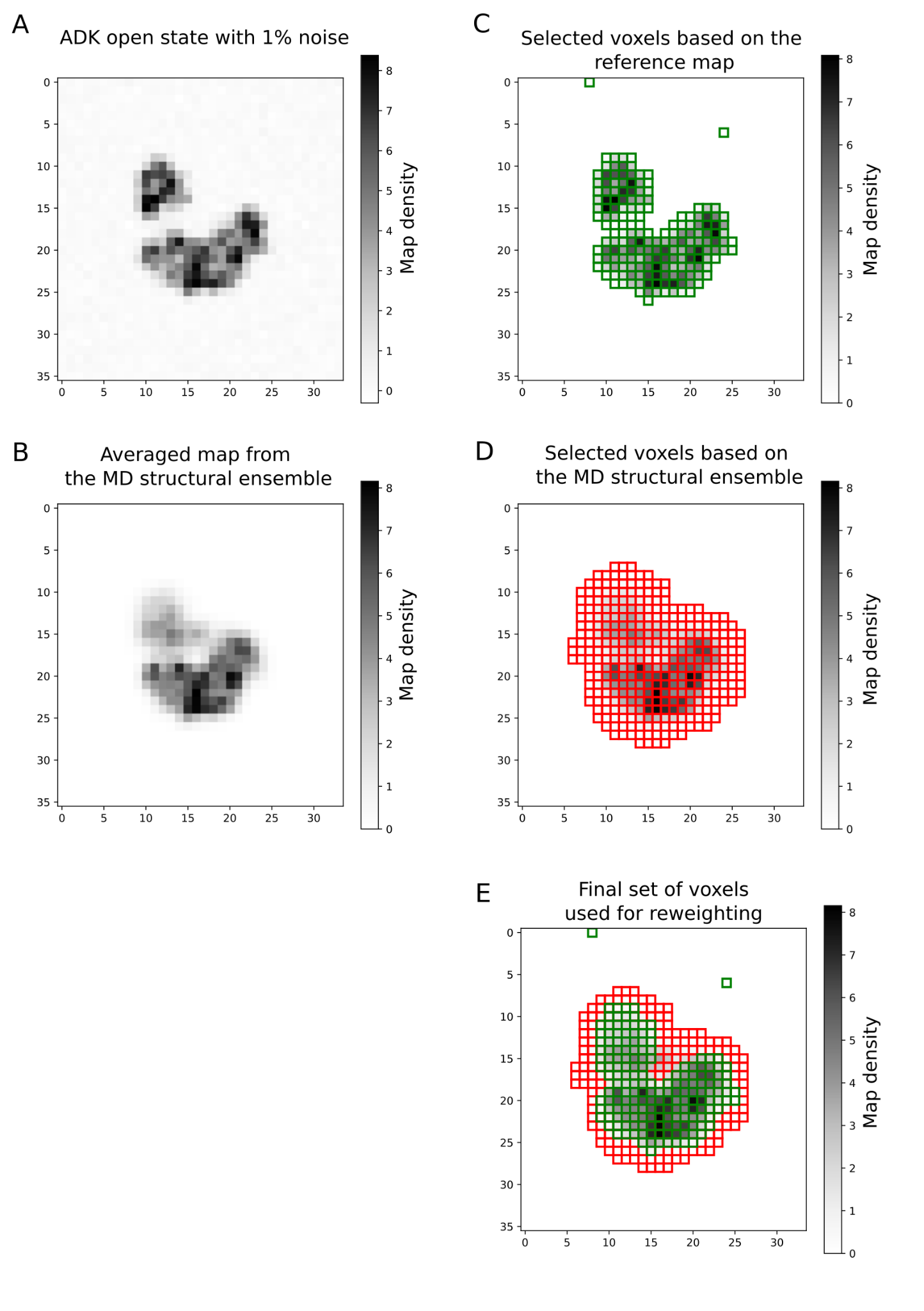
**

**Supplementary Figure 3.** The 2D cross-sections of the cryo-EM densities from the ADK open state with 1% noise level **(A)** and the averaged map from the corresponding MD structural ensemble **(B)**. **(C)** The cross-section from (**A**) with selected (in green) voxels that are above the noise level and will be used for reweighting. **(D)** The cross-section from (**B**) with selected (in red) voxels that are above the threshold level and will be used for reweighting. **(E)** The cross-section from (**B**) with a selected final set of voxels used for reweighting comprises ones selected based on cryo-EM reference density and from the MD ensemble.


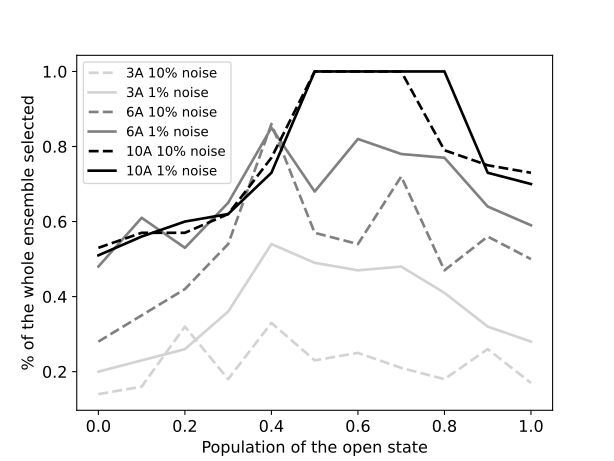


**Supplementary Figure 4.** Populations of the sub-ensembles obtained upon iterative reweighting of various ADK data sets.


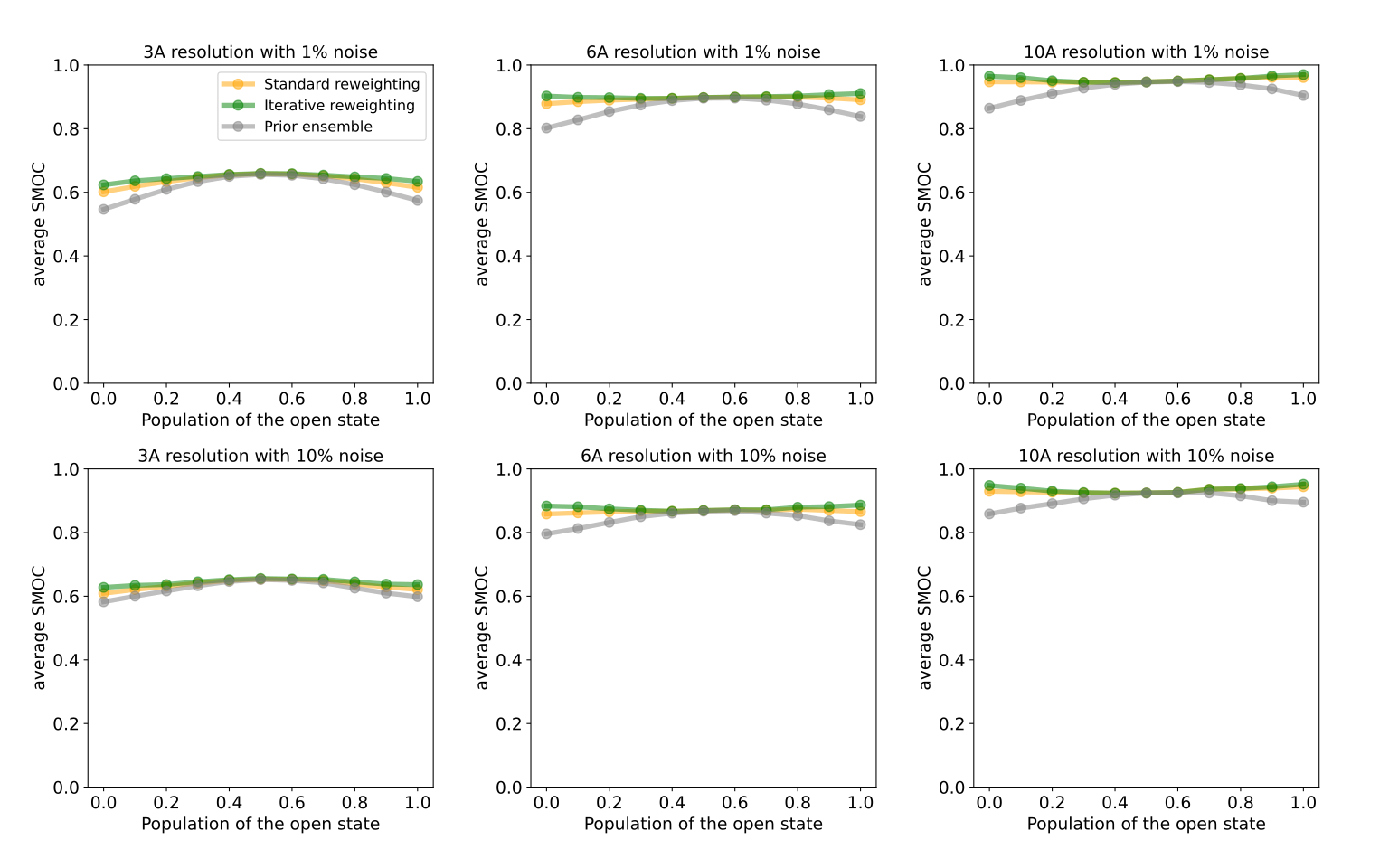


**Supplementary Figure 5.** Average SMOC score calculated between the reference map and the map generated from the structural ensemble upon reweighting for each ADK dataset. The datasets varied in resolution, noise level, and reference populations of the open state.


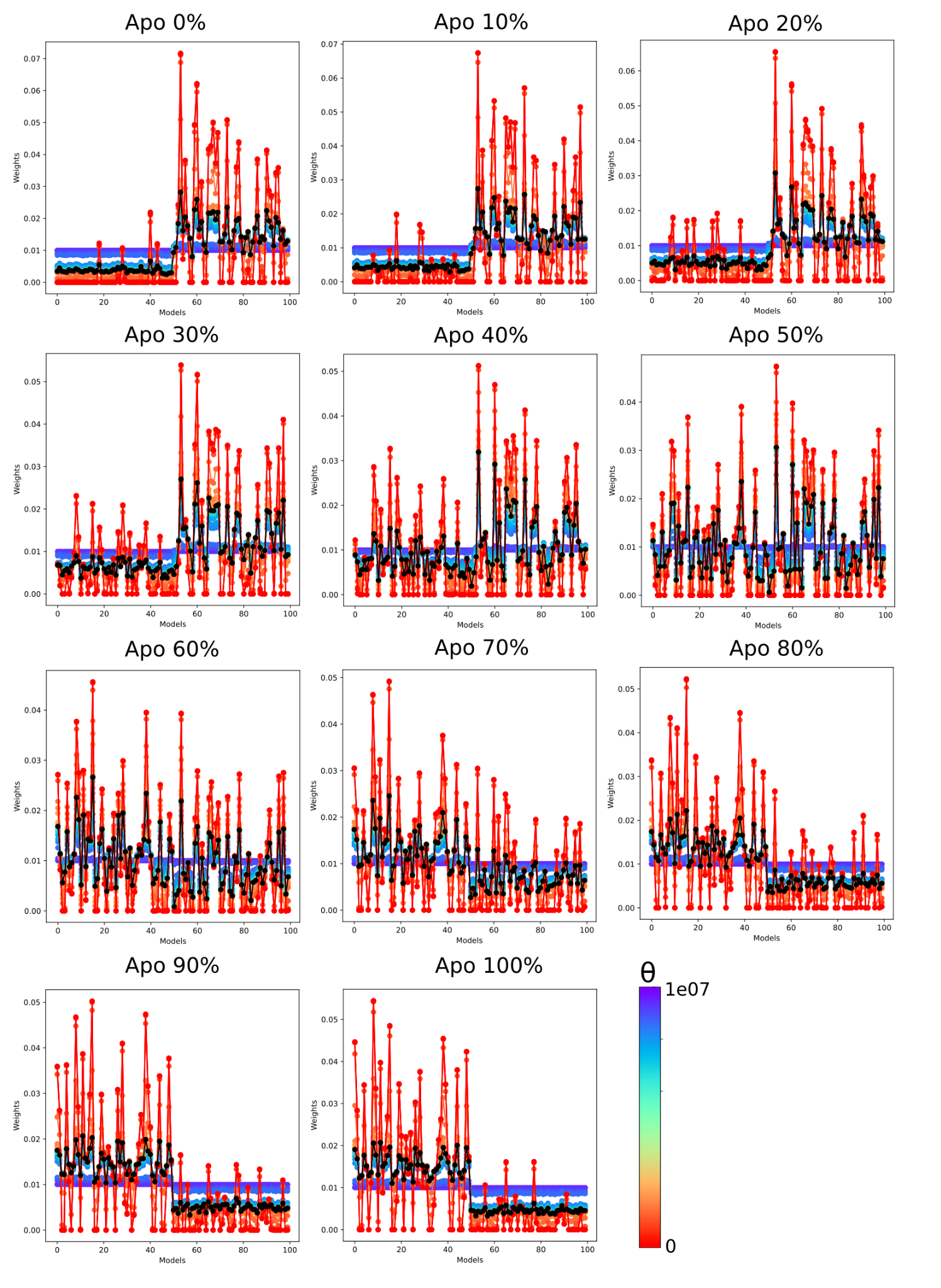


**Supplementary Figure 6**. Weights assigned to each model from the structural ensemble after the reweighting with the use of a 3 Å reference map with the different populations of the open state and a 10% noise level. The first 50 models represent the open conformation, while the last 50 models represent the closed conformation. Weights are presented for various θ values, with the optimal θ value shown in black.


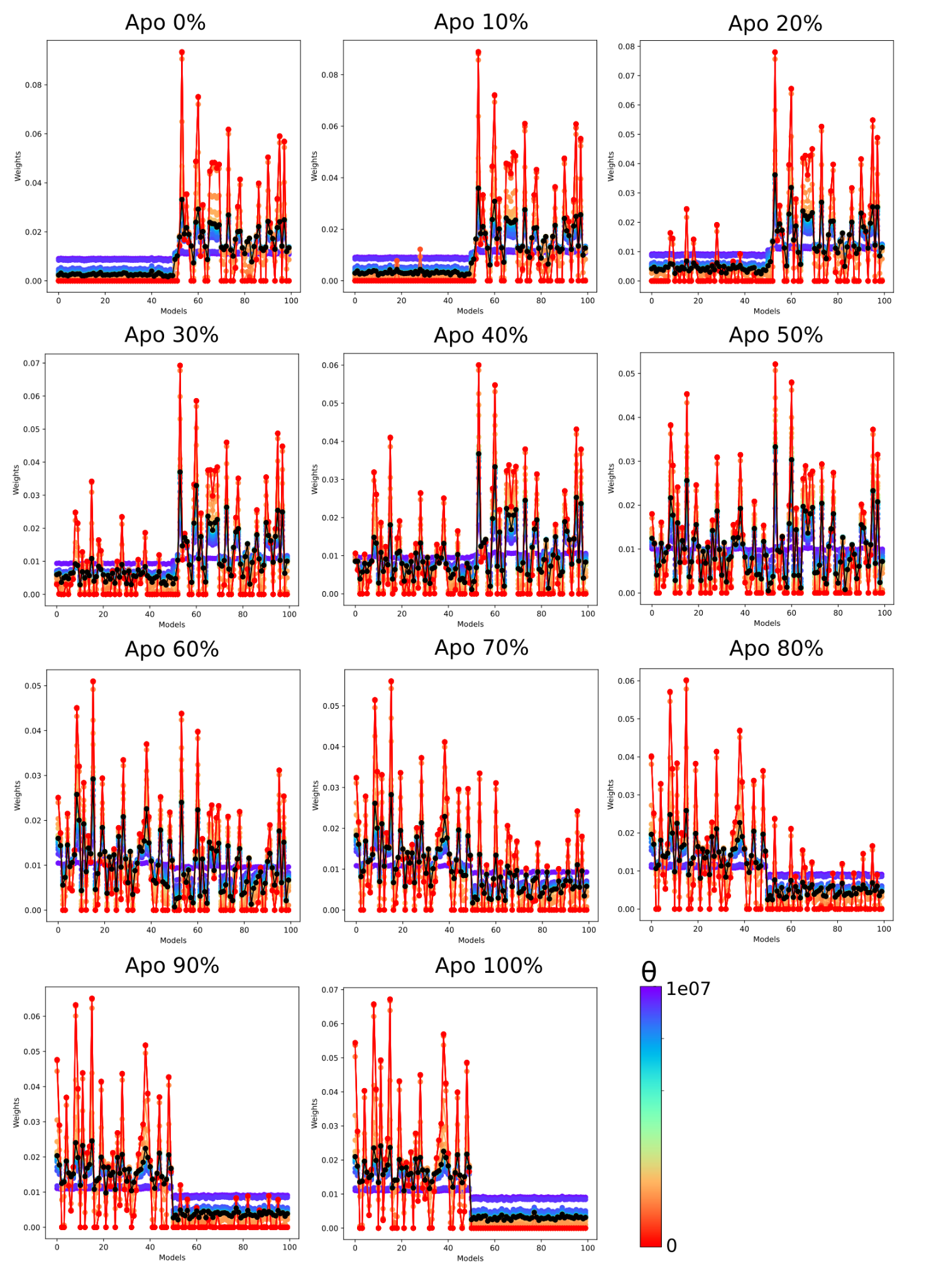


**Supplementary Figure 7**. Weights assigned to each model from the structural ensemble after the reweighting with the use of a 3 Å reference map with the different populations of the open state and a 1% noise level. The first 50 models represent the open conformation, while the last 50 models represent the closed conformation. Weights are presented for various θ values, with the optimal θ value shown in black.


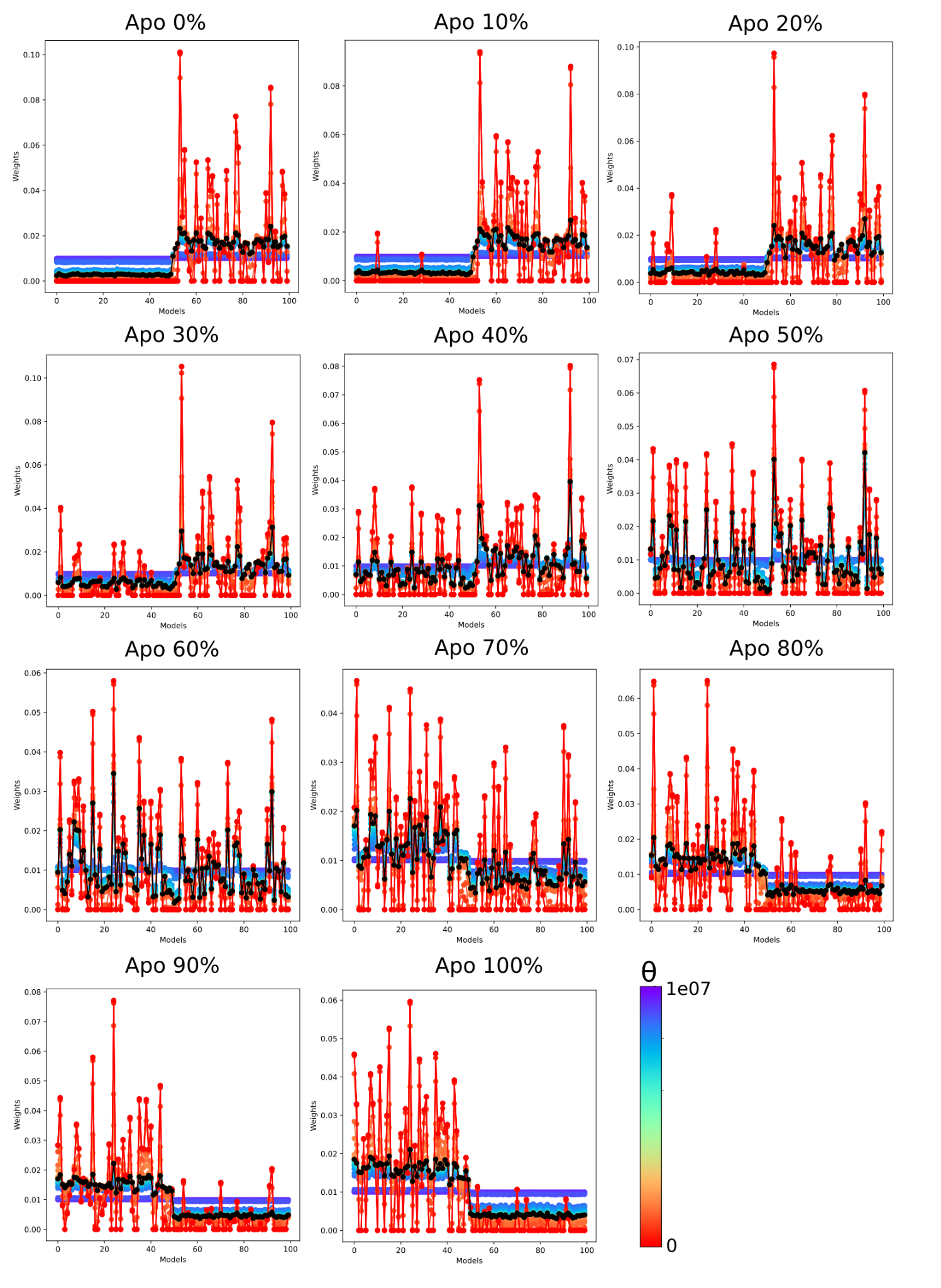


**Supplementary Figure 8**. Weights assigned to each model from the structural ensemble after the reweighting with the use of a 6 Å reference map with the different populations of the open state and a 10% noise level. The first 50 models represent the open conformation, while the last 50 models represent the closed conformation. Weights are presented for various θ values, with the optimal θ value shown in black.


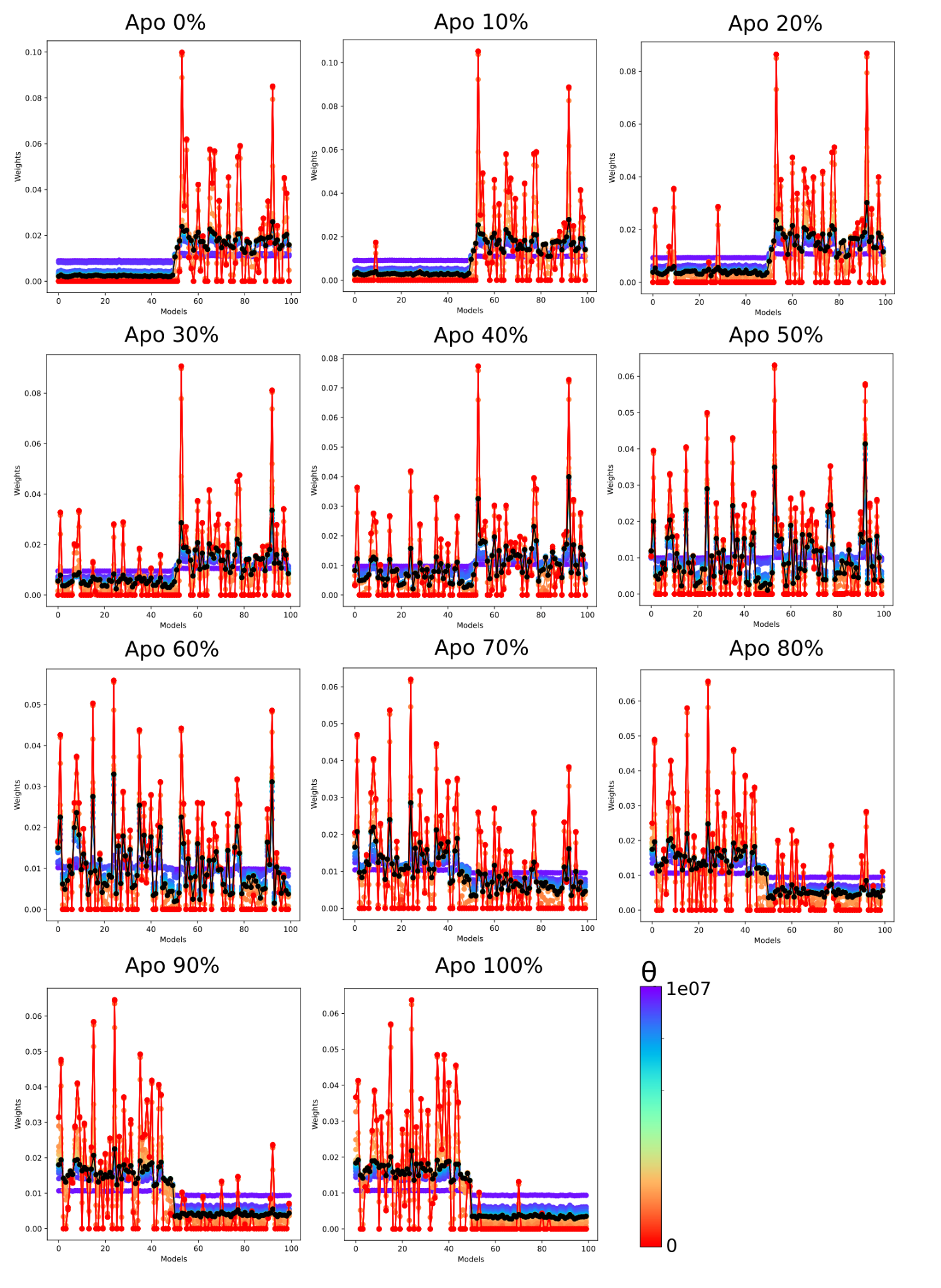


**Supplementary Figure 9**. Weights assigned to each model from the structural ensemble after the reweighting with the use of a 6 Å reference map with the different populations of the open state and a 1% noise level. The first 50 models represent the open conformation, while the last 50 models represent the closed conformation. Weights are presented for various θ values, with the optimal θ value shown in black.


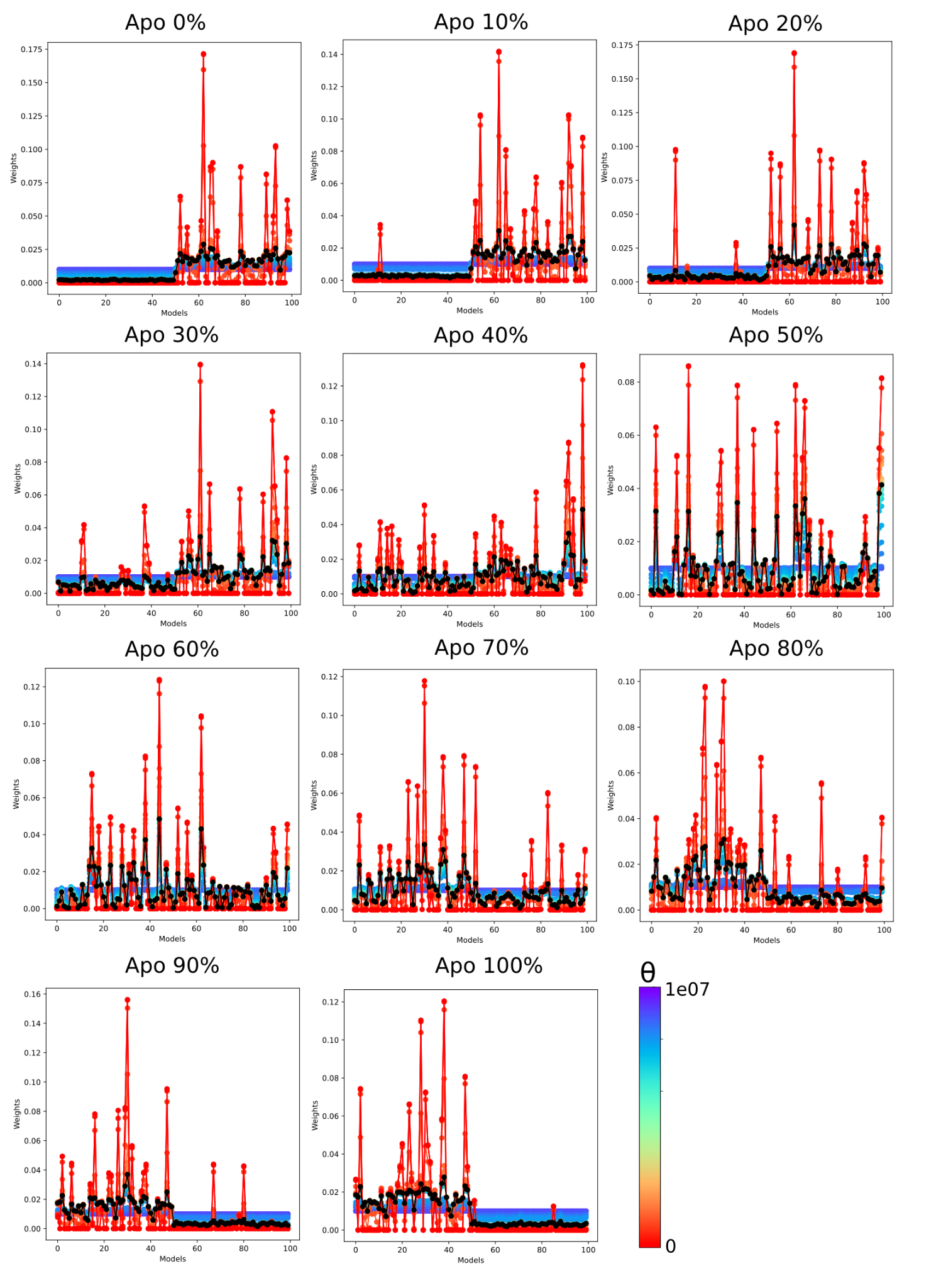


**Supplementary Figure 10.** Weights assigned to each model from the structural ensemble after the reweighting with the use of a 10 Å reference map with the different populations of the open state and a 10% noise level. The first 50 models represent the open conformation, while the last 50 models represent the closed conformation. Weights are presented for various θ values, with the optimal θ value shown in black.


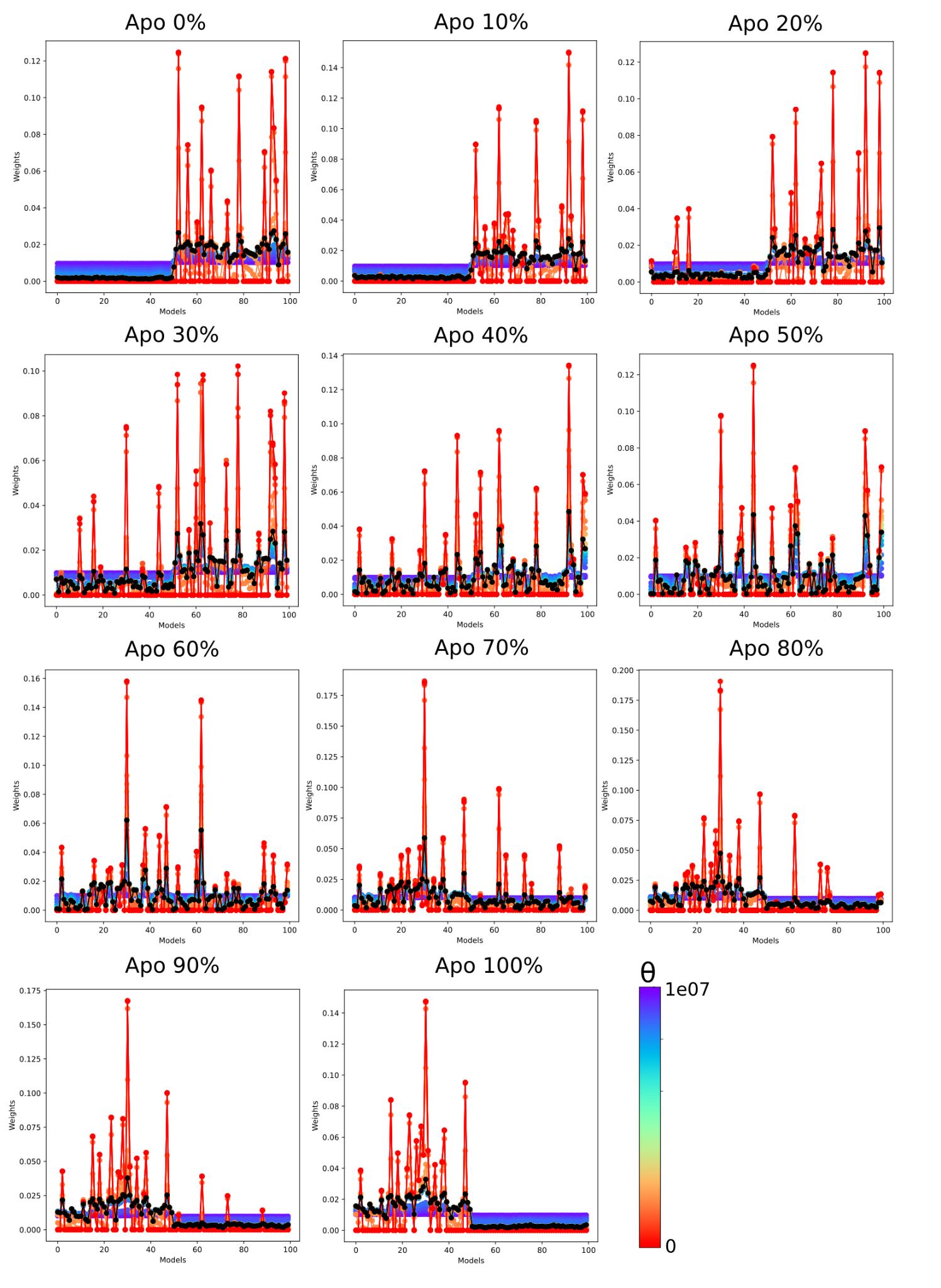


**Supplementary Figure 11**. Weights assigned to each model from the structural ensemble after the reweighting with the use of a 10 Å reference map with the different populations of the open state and a 1% noise level. The first 50 models represent the open conformation, while the last 50 models represent the closed conformation. Weights are presented for various θ values, with the optimal θ value shown in black.


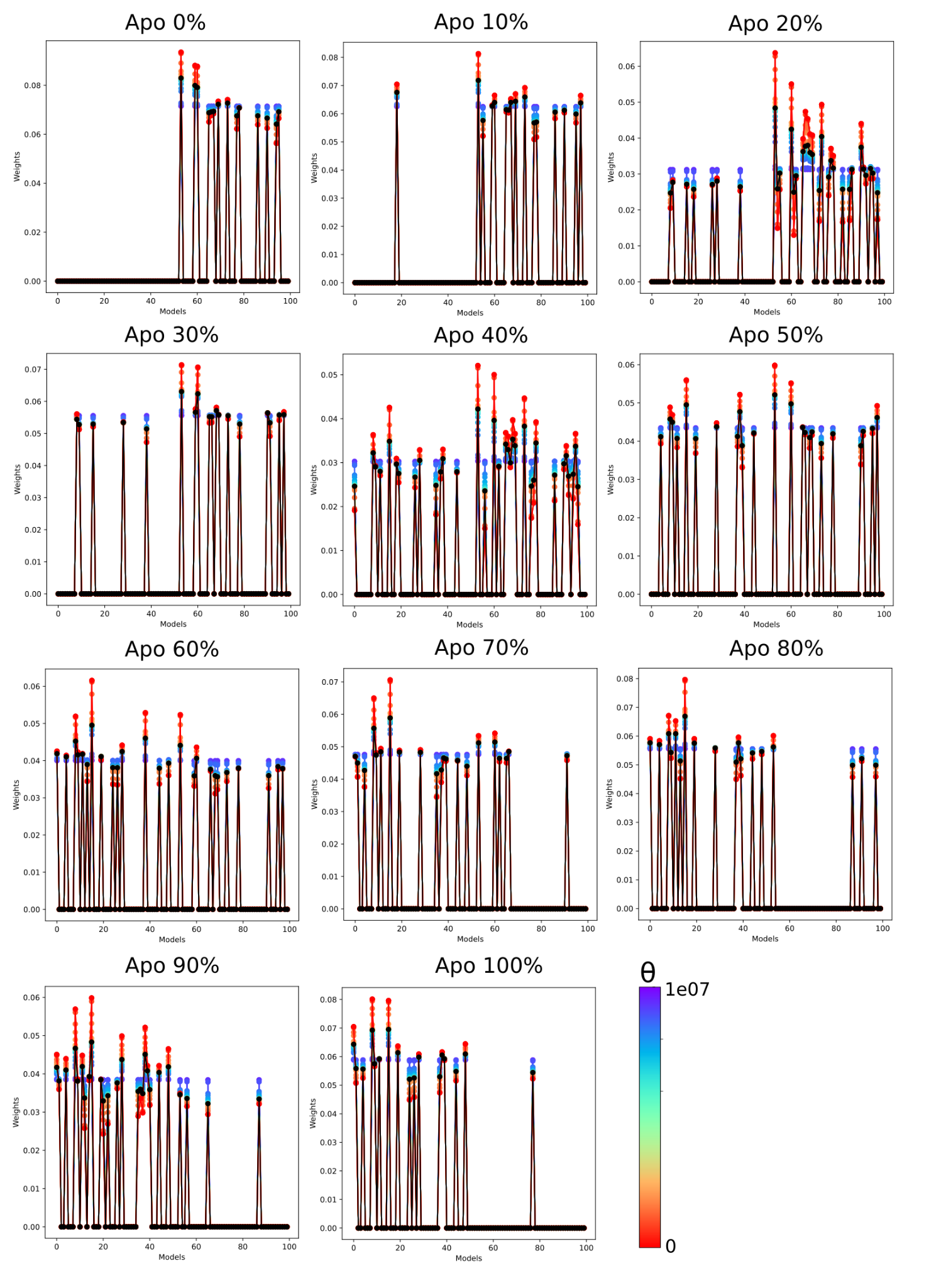


**Supplementary Figure 12**. Weights assigned to selected models from the structural ensemble after the iterative reweighting with the use of a 3 Å reference map with the different populations of the open state and a 10% noise level. The first 50 models represent the open conformation, while the last 50 models represent the closed conformation. Weights are presented for various θ values, with the optimal θ value shown in black.


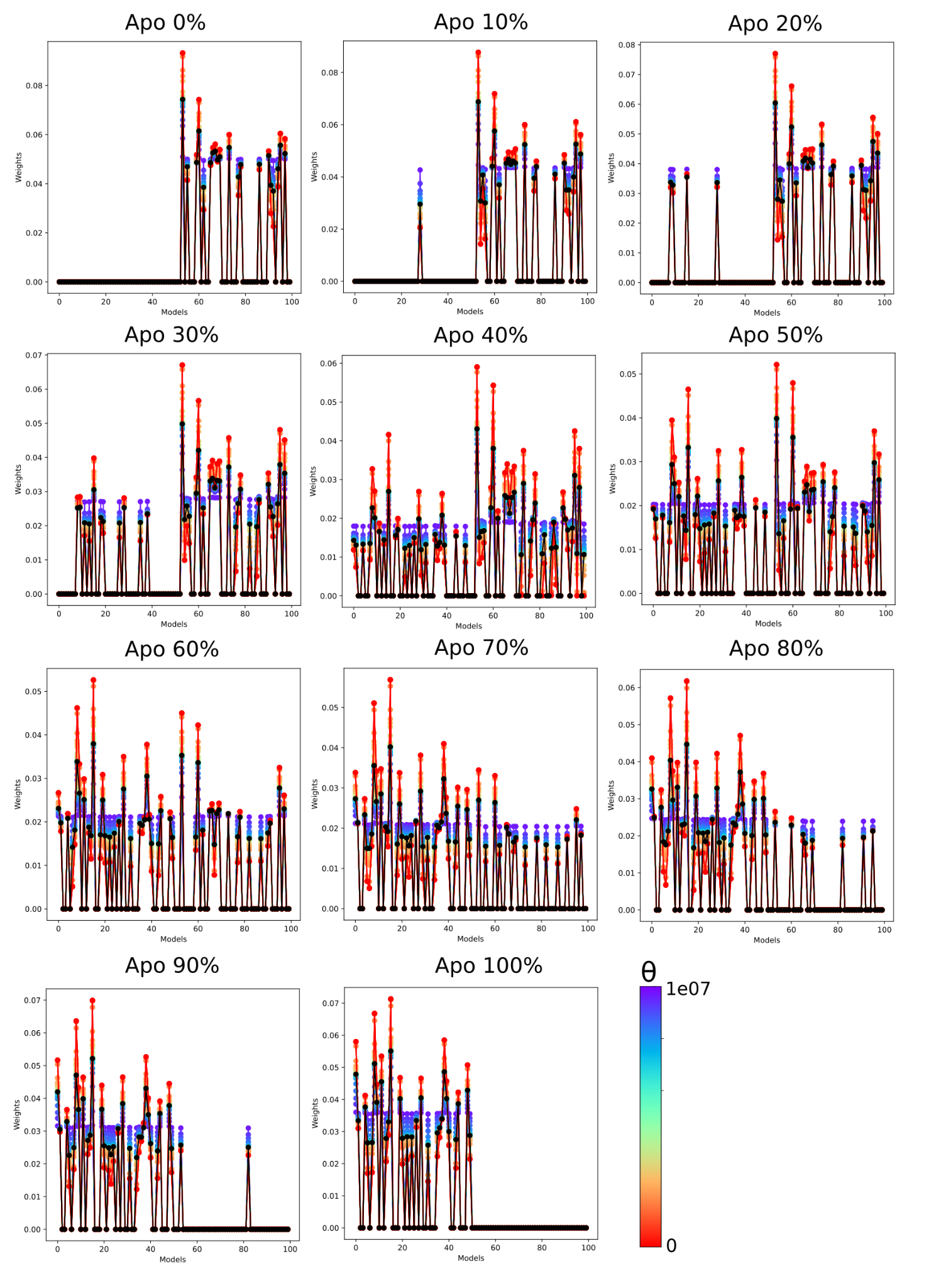


**Supplementary Figure 13**. Weights assigned to selected models from the structural ensemble after the iterative reweighting with the use of a 3 Å reference map with the different populations of the open state and a 1% noise level. The first 50 models represent the open conformation, while the last 50 models represent the closed conformation. Weights are presented for various θ values, with the optimal θ value shown in black.


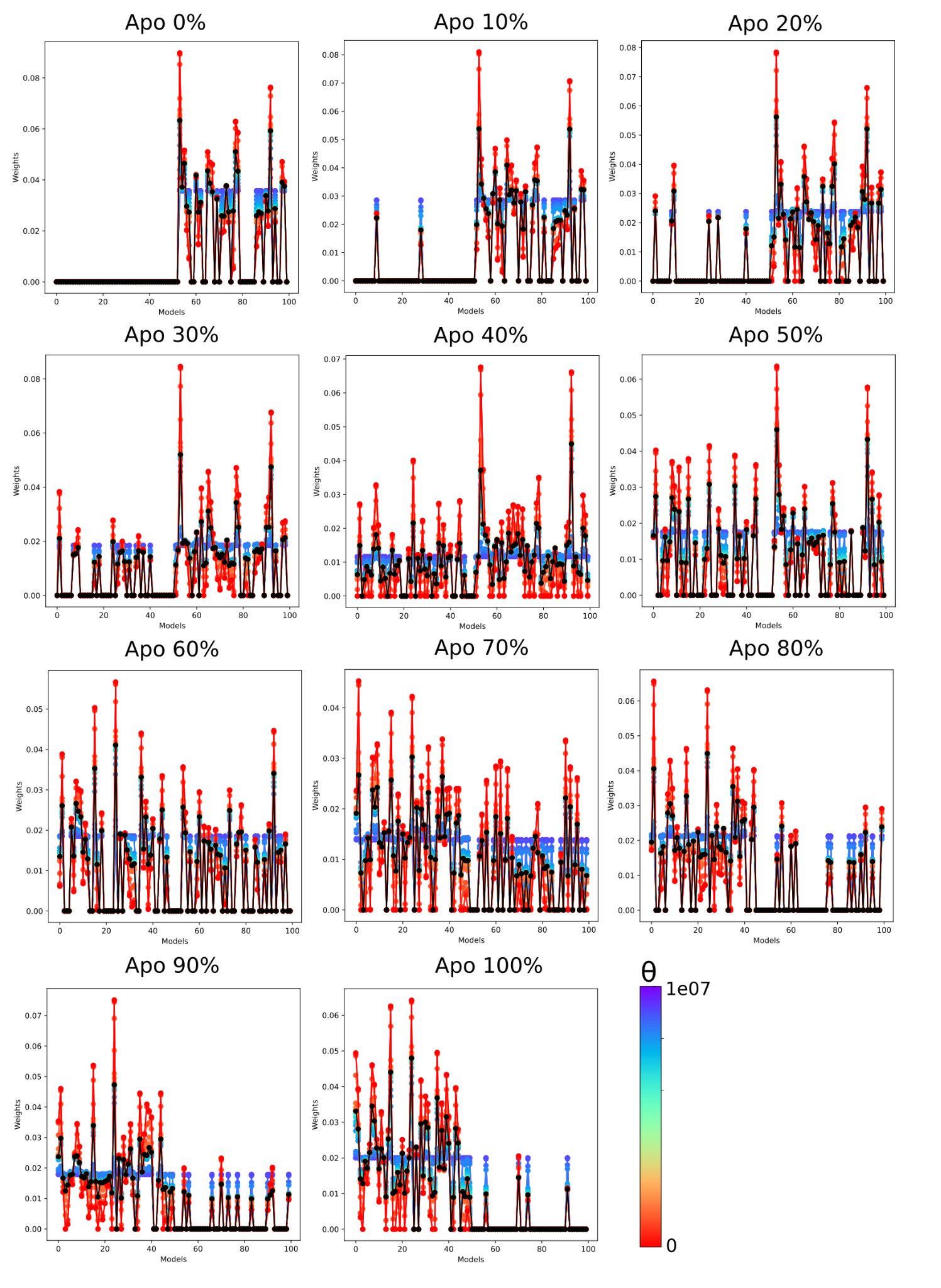


**Supplementary Figure 14**. Weights assigned to selected models from the structural ensemble after the iterative reweighting with the use of a 6 Å reference map with the different populations of the open state and a 10% noise level. The first 50 models represent the open conformation, while the last 50 models represent the closed conformation. Weights are presented for various θ values, with the optimal θ value shown in black.


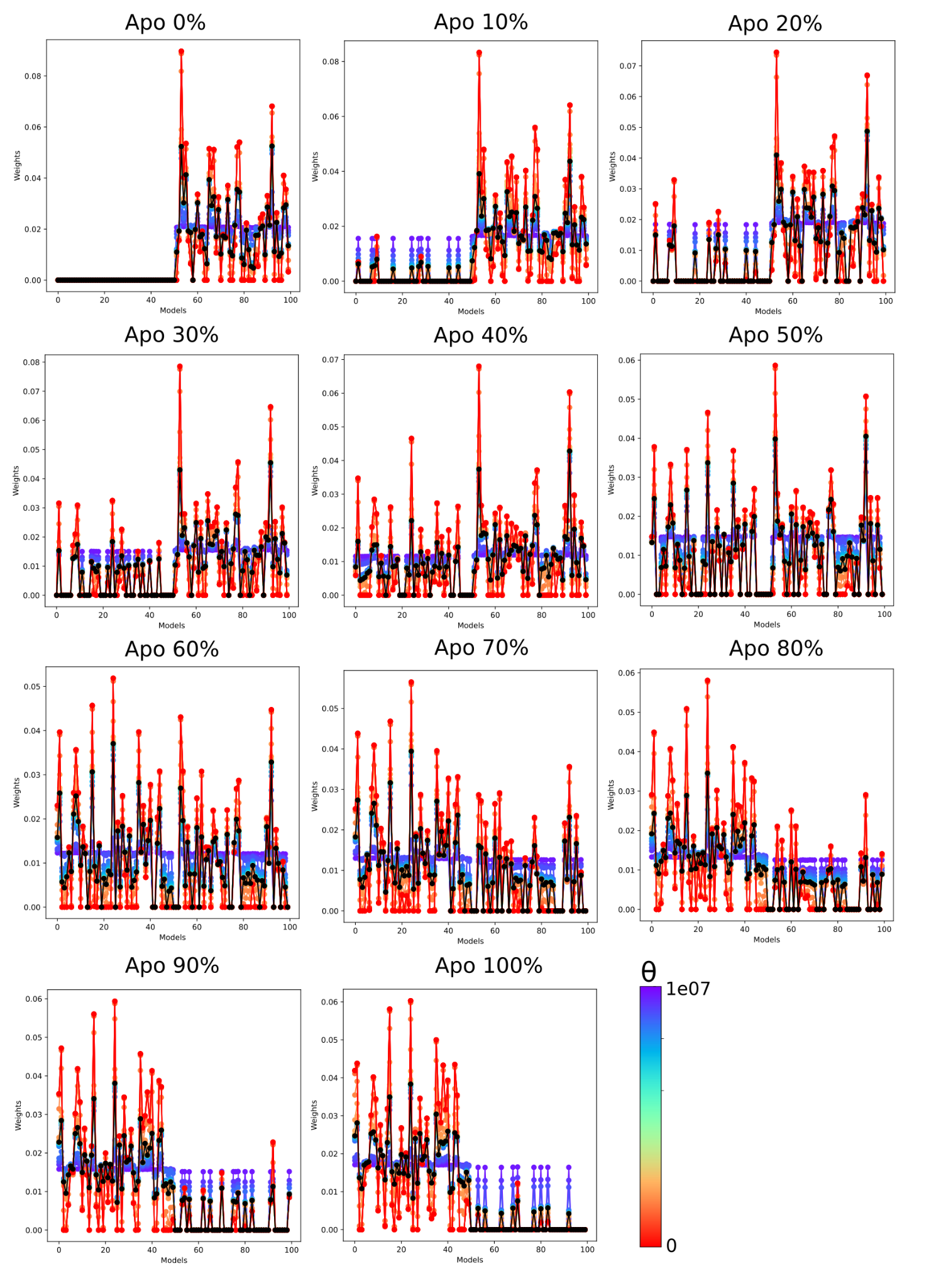


**Supplementary Figure 15**. Weights assigned to selected models from the structural ensemble after the iterative reweighting with the use of a 6 Å reference map with the different populations of the open state and a 1% noise level. The first 50 models represent the open conformation, while the last 50 models represent the closed conformation. Weights are presented for various θ values, with the optimal θ value shown in black.


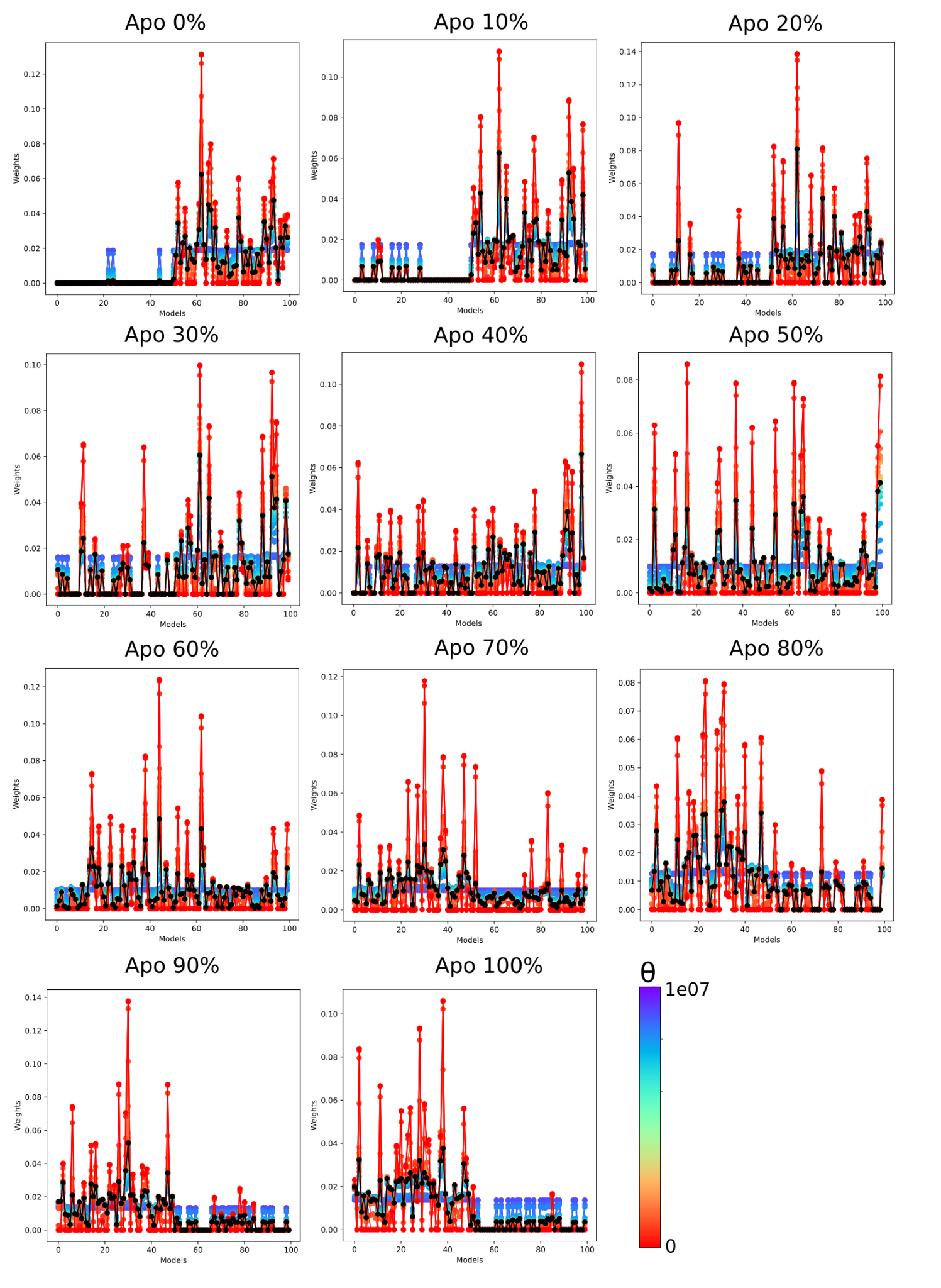


**Supplementary Figure 16**. Weights assigned to selected models from the structural ensemble after the iterative reweighting with the use of a 10 Å reference map with the different populations of the open state and a 10% noise level. The first 50 models represent the open conformation, while the last 50 models represent the closed conformation. Weights are presented for various θ values, with the optimal θ value shown in black.


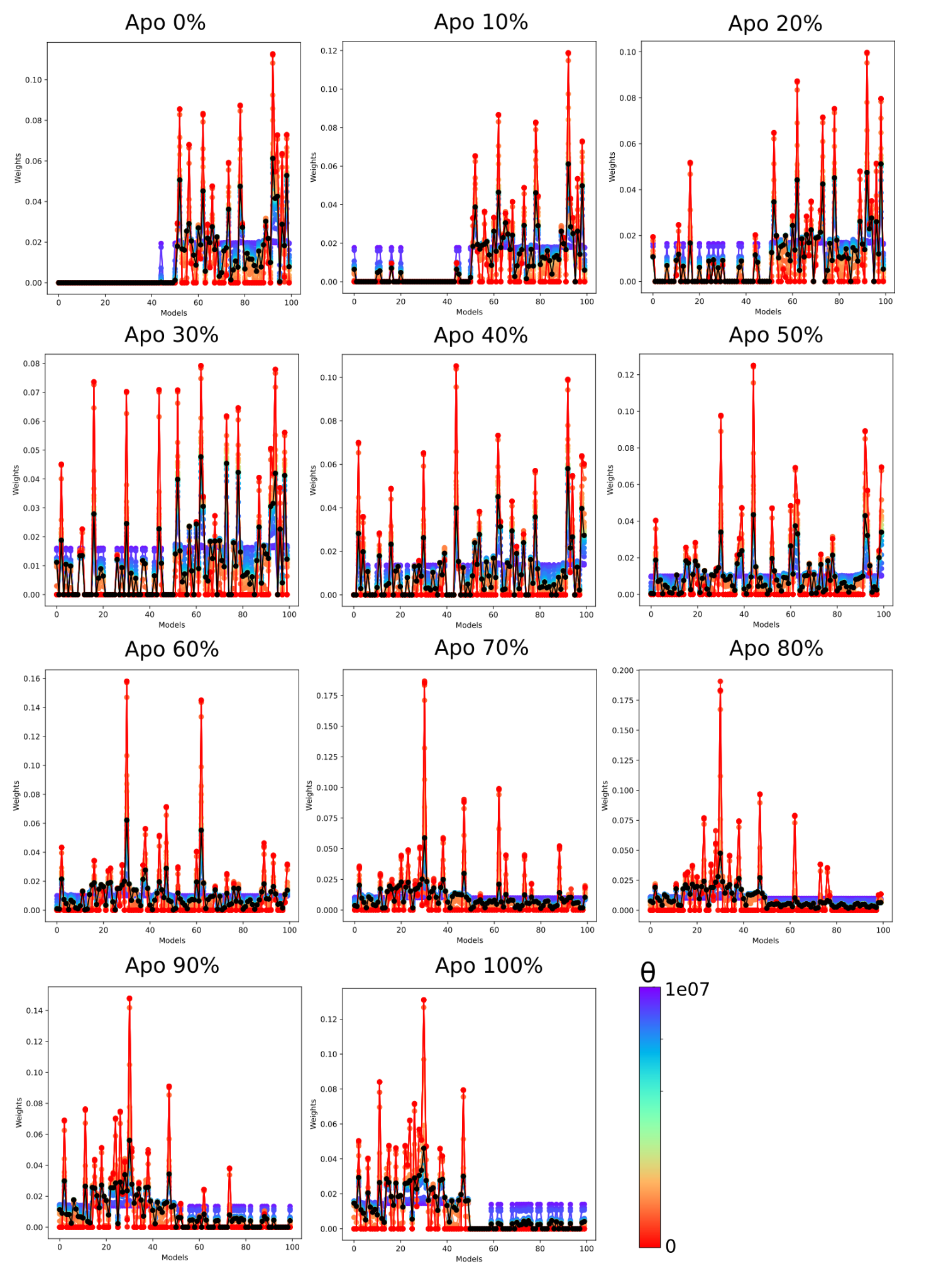


**Supplementary Figure 17**. Weights assigned to selected models from the structural ensemble after the iterative reweighting with the use of a 10 Å reference map with the different populations of the open state and a 1% noise level. The first 50 models represent the open conformation, while the last 50 models represent the closed conformation. Weights are presented for various θ values, with the optimal θ value shown in black.


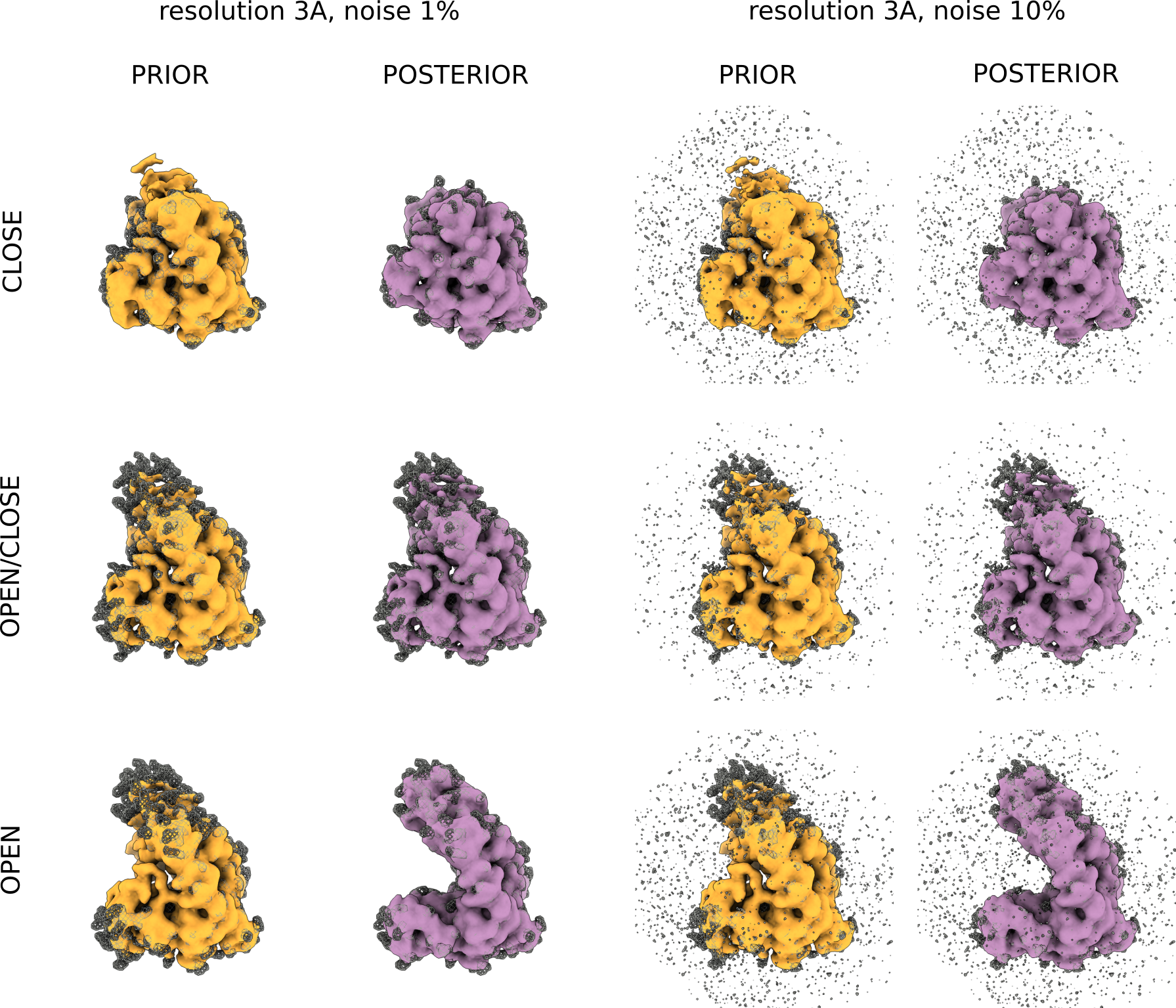


**Supplementary Figure 18.** Visual comparison of the 3 Å reference maps (displayed in grey mesh) with maps generated from the structural ensemble prior (in orange) and posterior (in purple) reweighting. The threshold for map visualisation is set at two standard deviations of the map density.


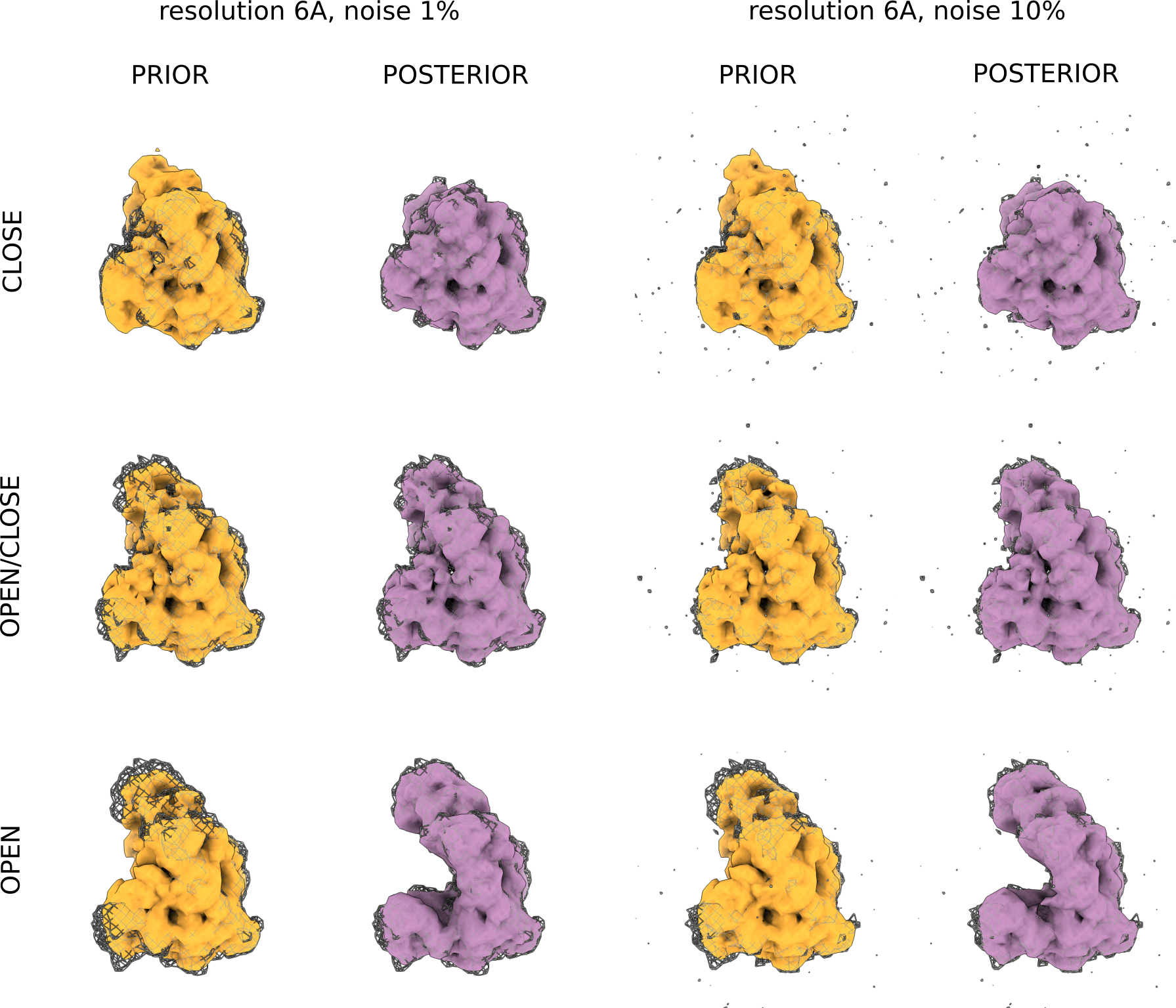


**Supplementary Figure 19.** Visual comparison of the 6 Å reference maps (displayed in grey mesh) with maps generated from the structural ensemble prior (in orange) and posterior (in purple) reweighting. The threshold for map visualisation is set at two standard deviations of the map density.


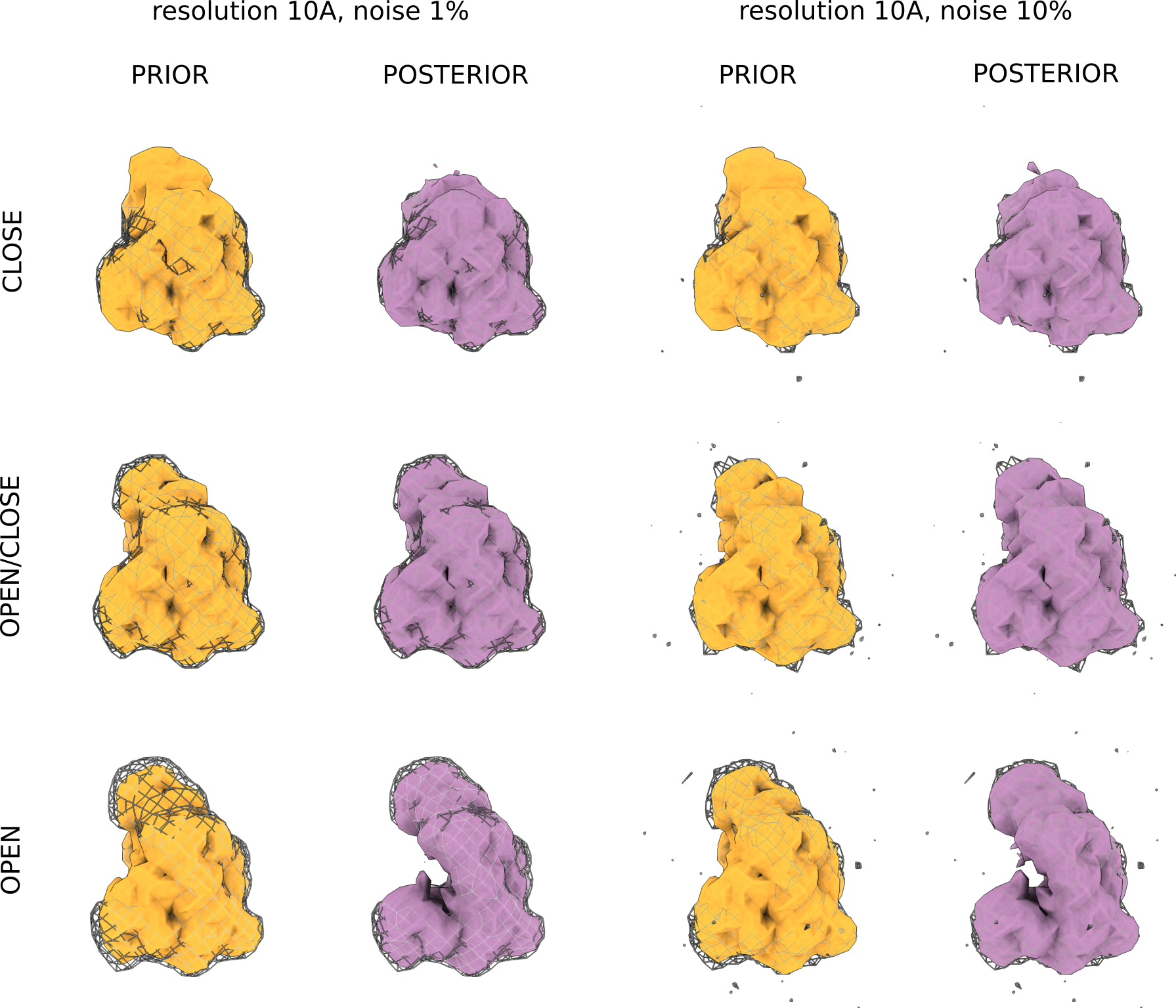


**Supplementary Figure 20.** Visual comparison of the 10 Å reference maps (displayed in grey mesh) with maps generated from the structural ensemble prior (in orange) and posterior (in purple) reweighting. The threshold for map visualisation is set at two standard deviations of the map density.

***
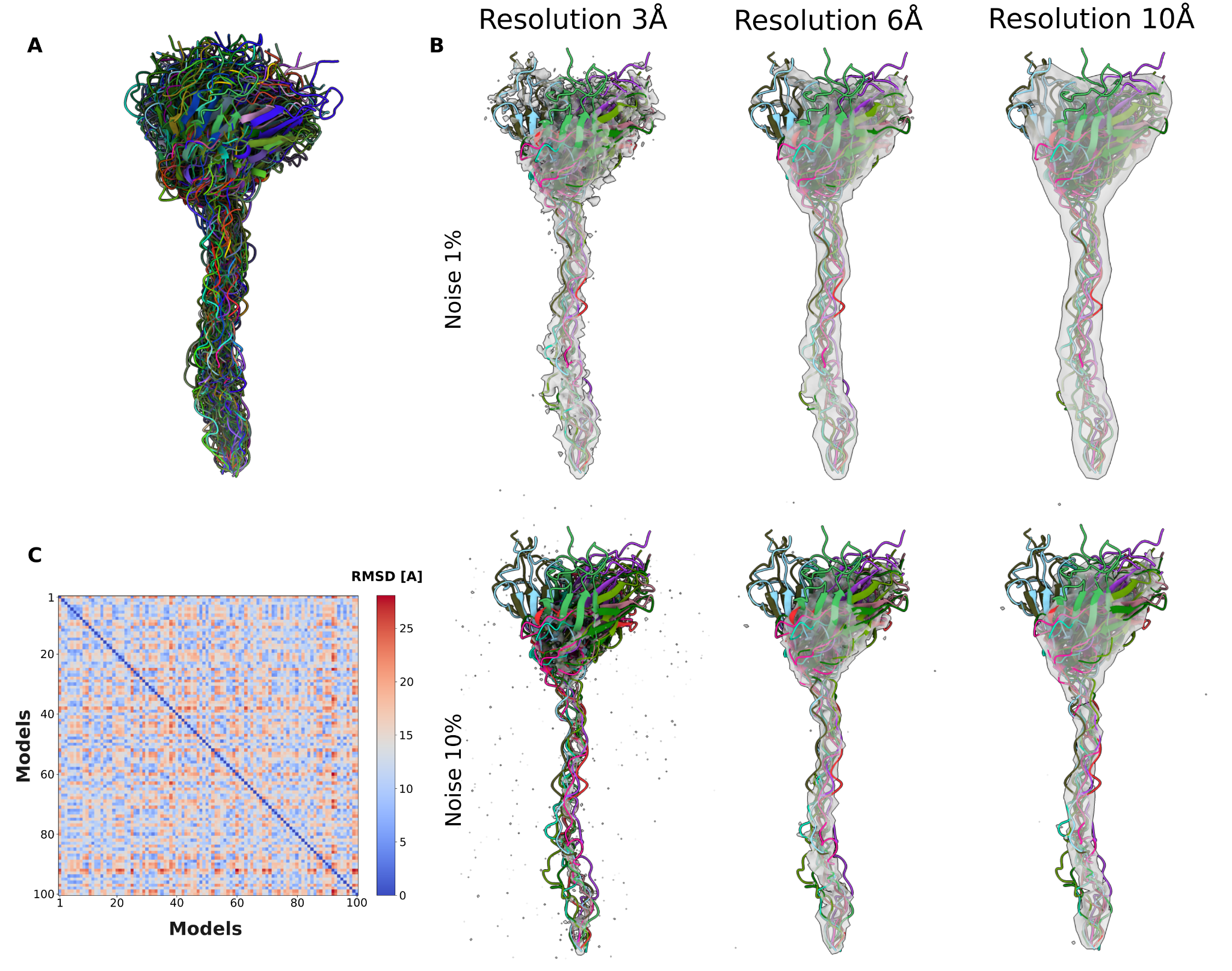
***

**Supplementary Figure 21. (A)** FLN5-6 structural ensemble, encompassing 100 structures, selected from a long MD simulation. **(B)** Examples of ten random FLN5-6 nascent chain structures chosen from the MD ensemble **(A)** used to generate the reference density map at different resolutions (3, 6, and 10 Å) and noise levels (1% or 10%). Each structure is depicted in a different colour and combines the FLN5-6 nascent chain that is composed of N-terminal folded FLN5 followed by 31 amino acids of the subsequent FLN6 domain and a C-terminal SecM stalling sequence that is covalently attached to the tRNA at peptidyl transferase centre of 70S ribosome. All maps are depicted at a level equal to three times the standard deviation of the noise distribution. **(C)** RMSD matrix calculated for the FLN5 structural ensemble without structure superimposition.


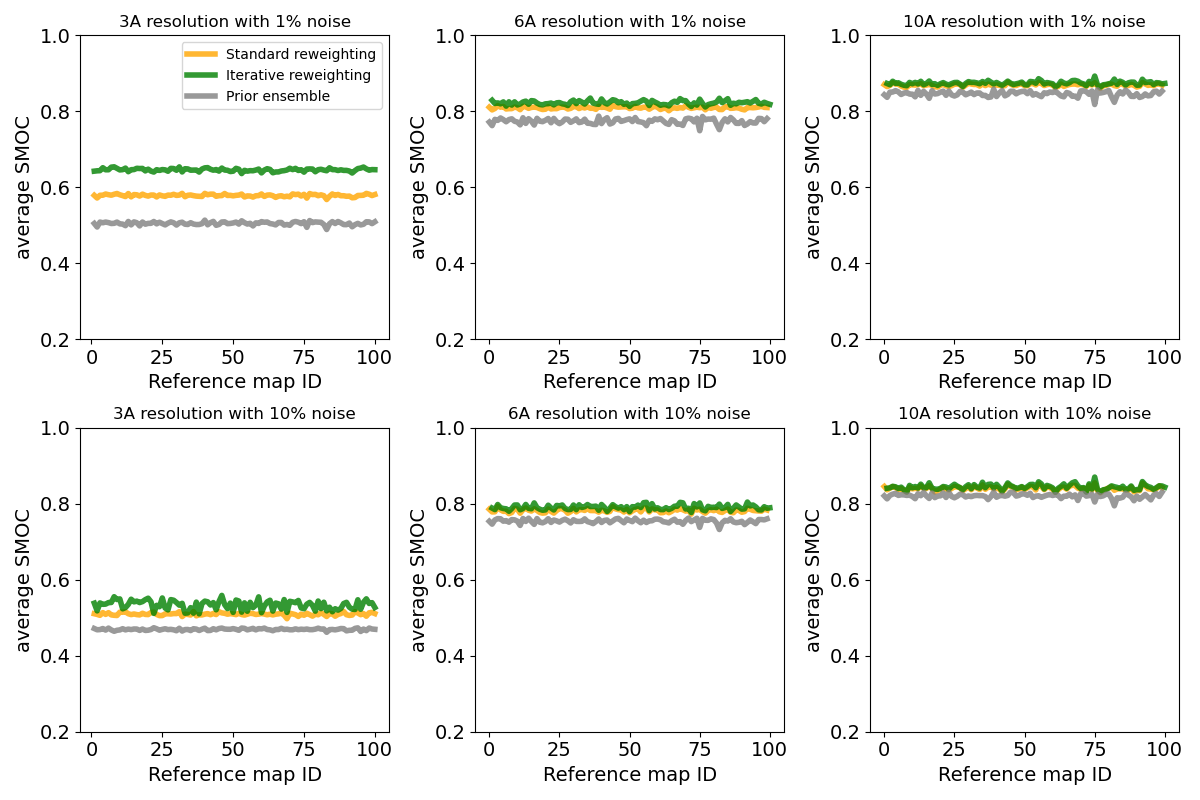


**Supplementary Figure 22**. Average SMOC score calculated between the reference map and the maps generated from the structural ensemble upon standard (in orange) and iterative (in green) reweighting for each FLN5 dataset. The datasets varied in resolution and noise level.


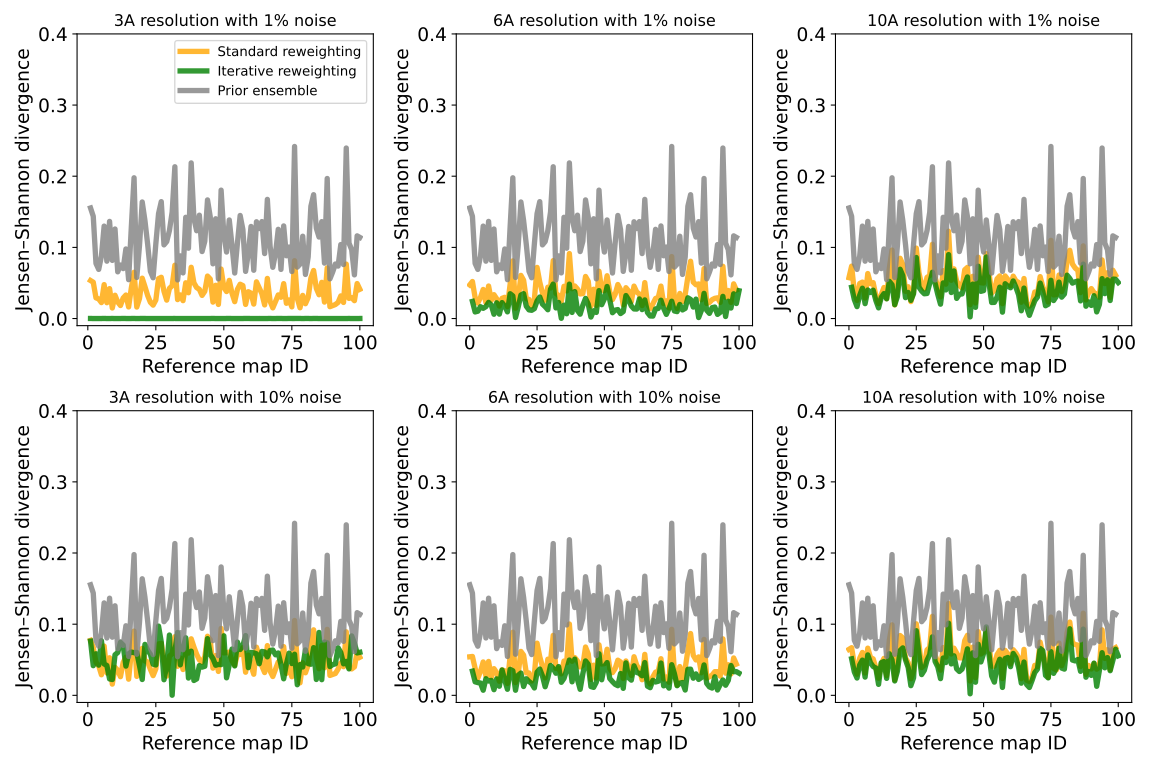


**Supplementary Figure 23**. Jensen-Shannon divergence calculated between the MD ensemble (before and after standard (in orange) and iterative (in green) reweighting) and the ensemble consisting of 10 structures used for map generation.


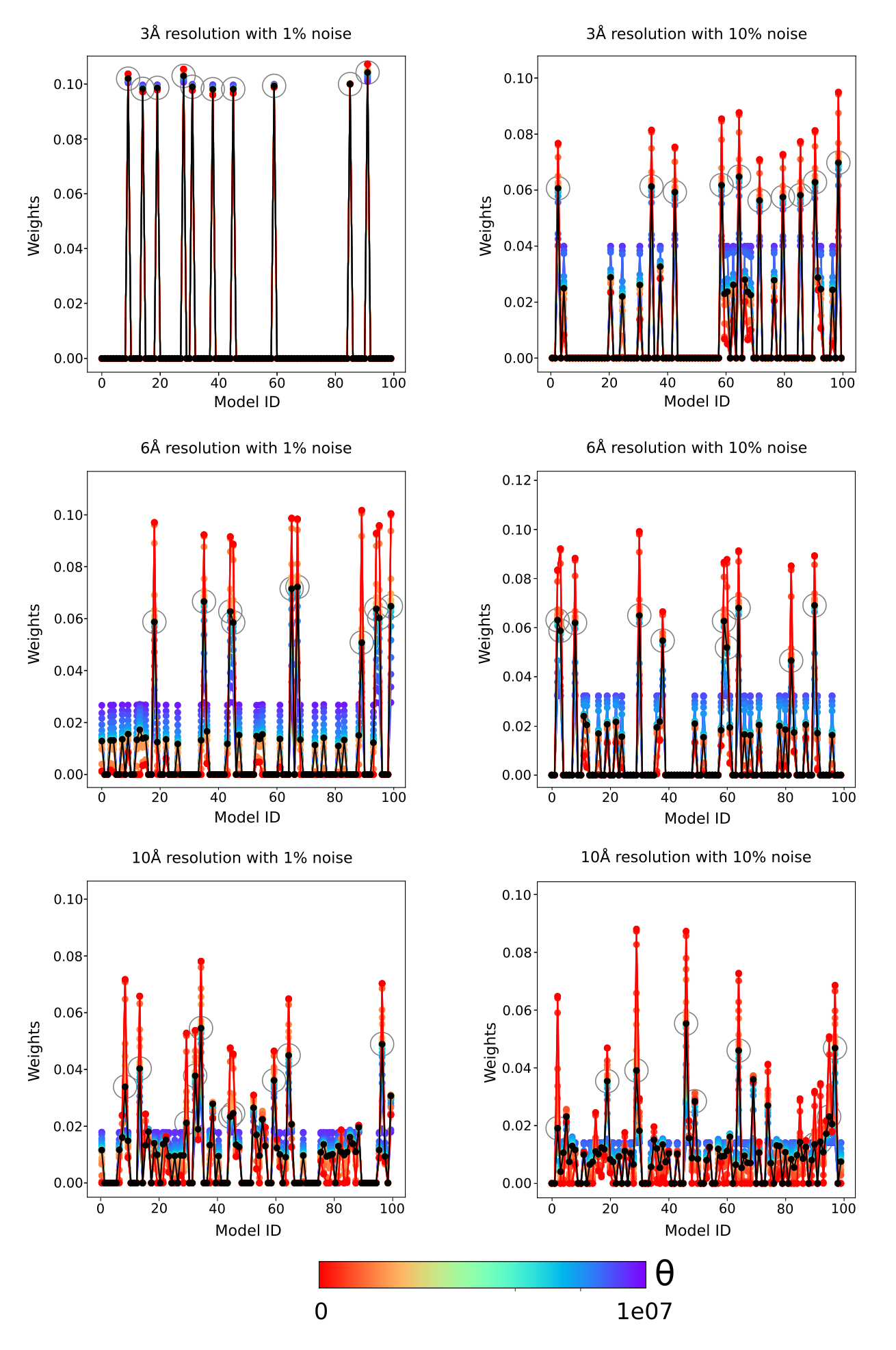


**Supplementary Figure 24**. Weights obtained upon cryoENsemble iterative reweighting of the FLN5-6 nascent chain dataset that corresponds to the systems depicted on Fig. 3B. Weights are calculated with different theta (θ) values ranging from 0 to 10^7^, and with black lines, we depict optimal weights selected based on the L-curve analysis. Additionally, weights corresponding to the ten models used to generate the reference map are circled.


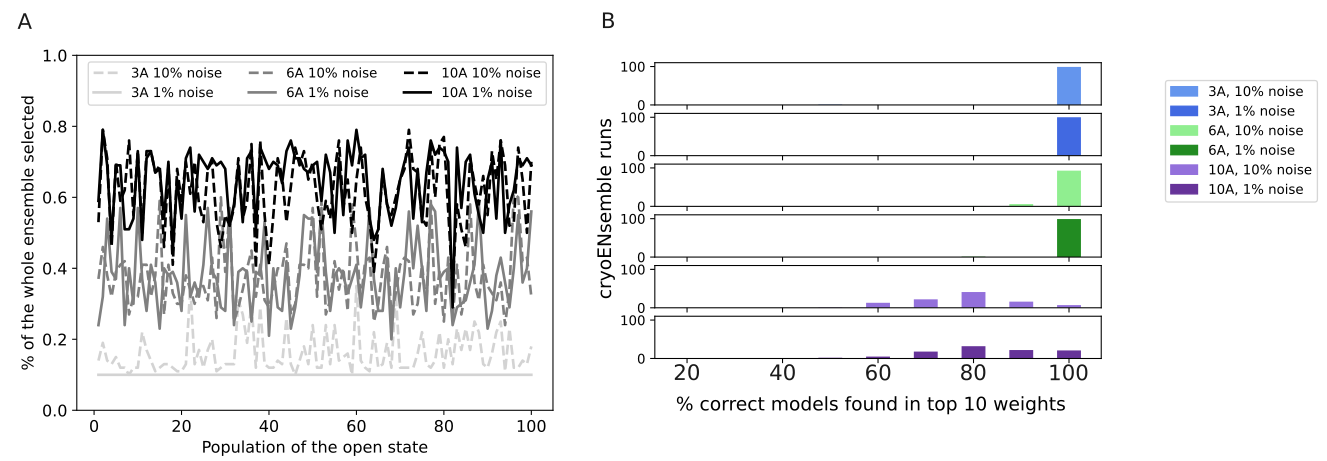


**Supplementary Figure 25. (A)** The populations of the sub-ensembles obtained upon iterative reweighting of various FLN5-6 data sets. **(B)** Histograms presenting the number of correct models found in the top 10 models ranked by weights in standard cryoENsemble runs for each NC dataset.


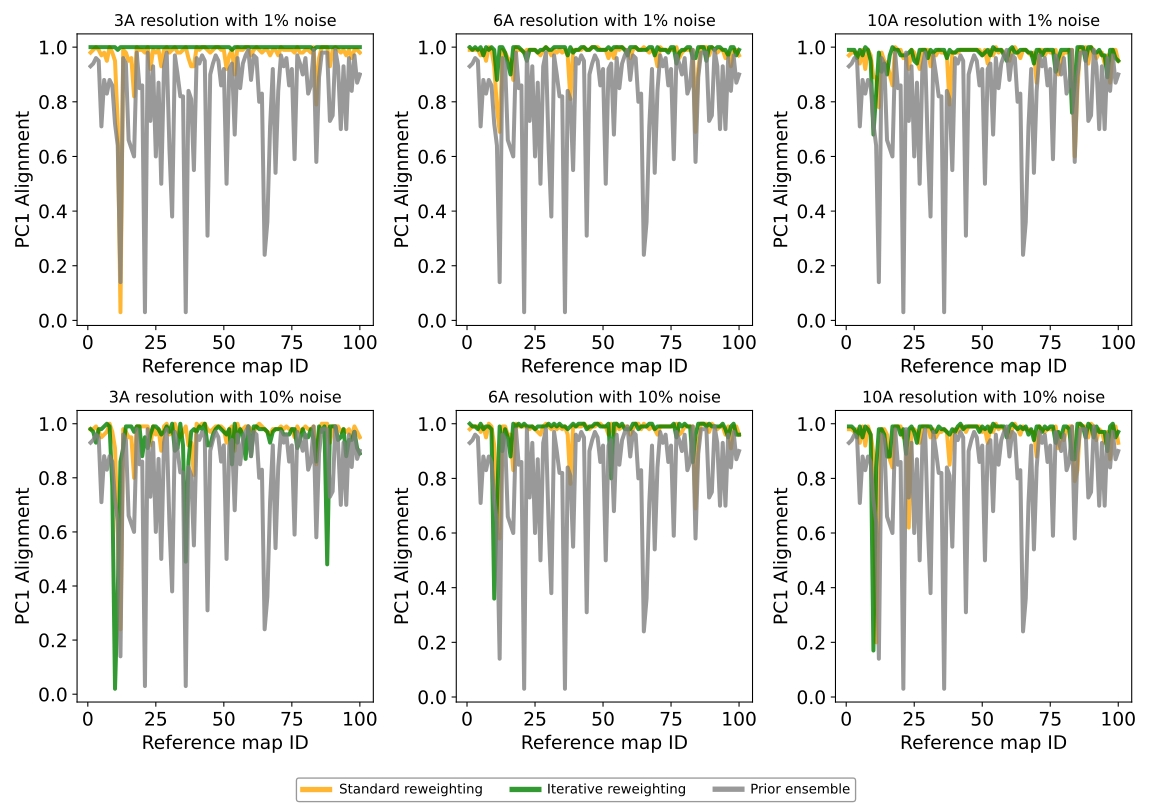


**Supplementary Figure 26**. Alignments of the first principal component (PC1) obtained from the target ensemble compared to the PC1 from the prior ensemble (in grey), and the posterior ensembles obtained with standard reweighting (in orange) or iterative reweighting (in green).


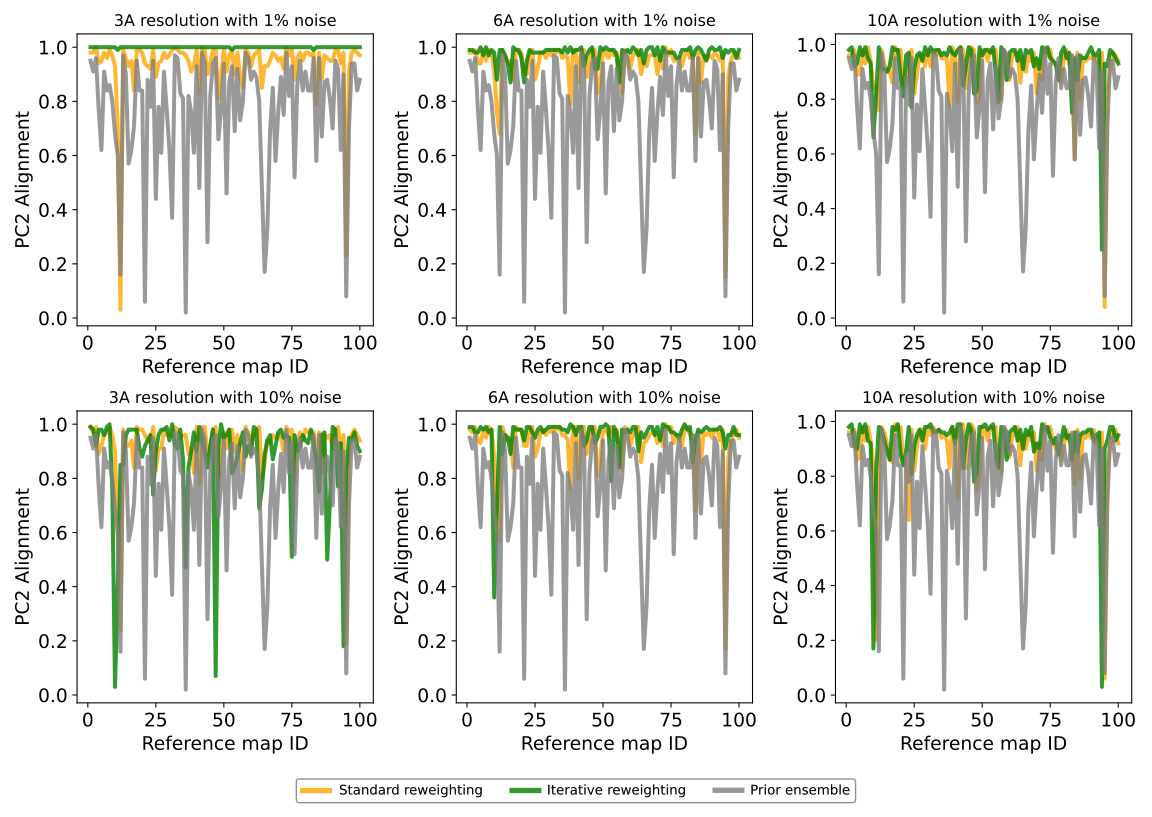


**Supplementary Figure 27**. Alignments of the second principal component (PC2) obtained from the target ensemble compared to the PC2 from the prior ensemble (in grey), and the posterior ensembles obtained with standard reweighting (in orange) or iterative reweighting (in green).


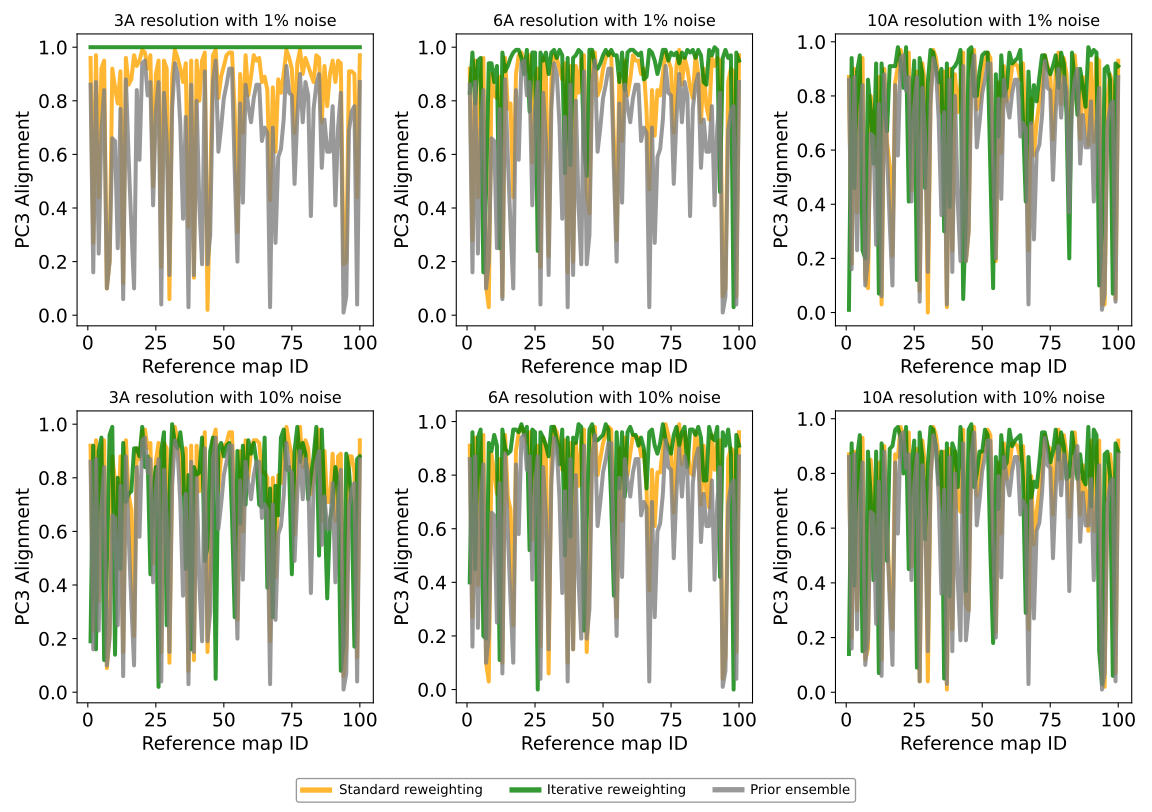


**Supplementary Figure 28**. Alignments of the third principal component (PC3) obtained from the target ensemble compared to the PC3 from the prior ensemble (in grey), and the posterior ensembles obtained with standard reweighting (in orange) or iterative reweighting (in green).

**
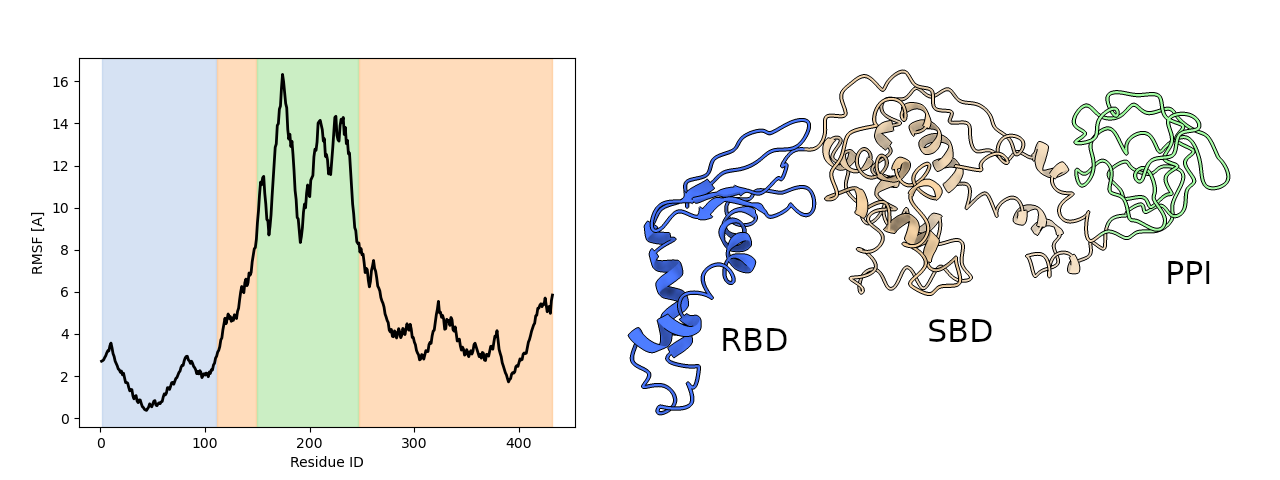
**

**Supplementary Figure 29**. Root-mean-square fluctuation (RMSF) calculated from a long all-atom MD simulation of the TF bound to the ribosome. Regions corresponding to each domain of the TF are marked with colours: the ribosome binding domain (RBD) in blue, the peptidyl-prolyl *cis-trans* isomerase (PPI) domain in green and the subtract binding domain (SBD) in orange.


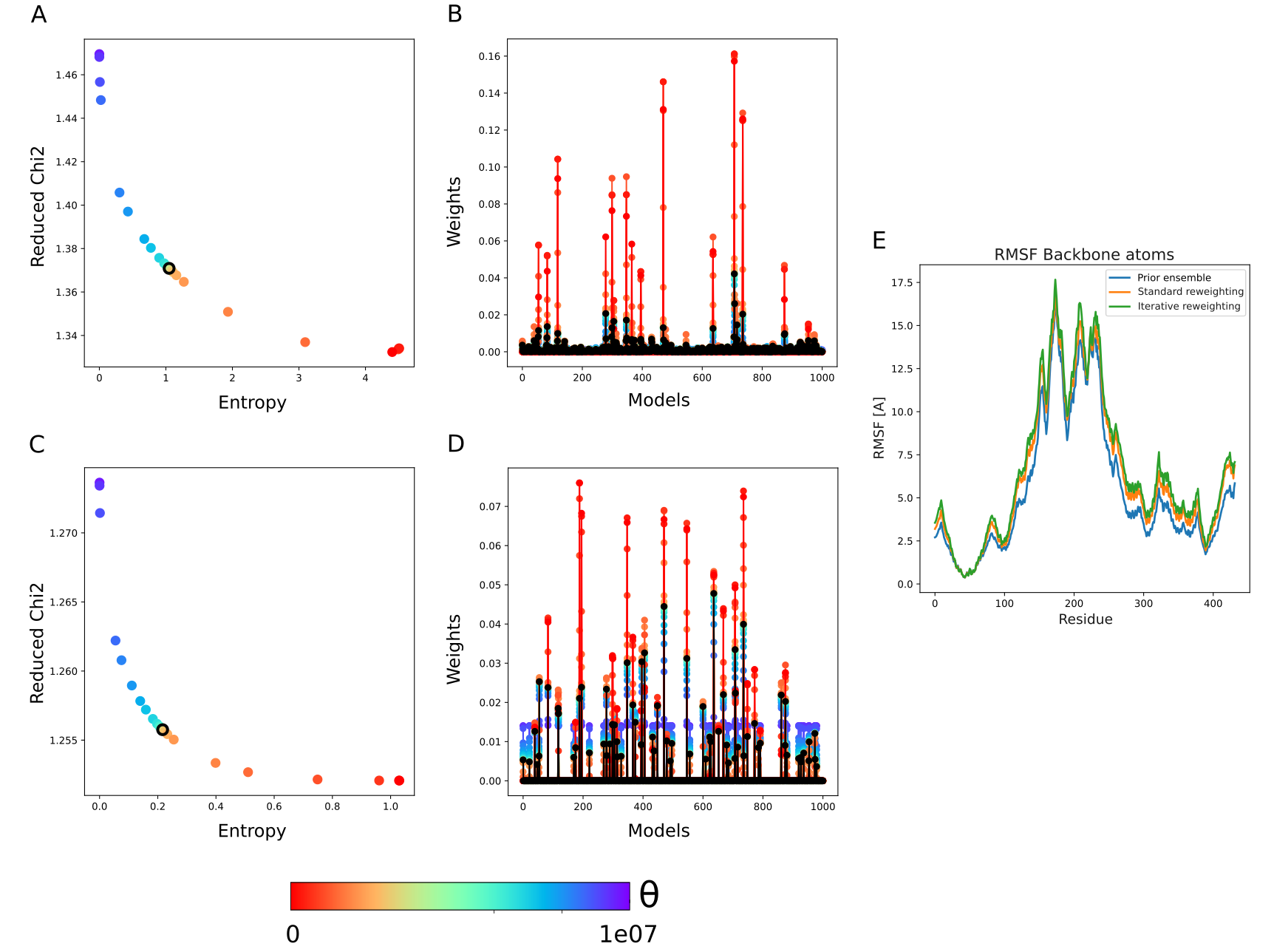


**Supplementary Figure 30**. Results of the trigger factor dataset reweighting. The L-curve analysis selects the optimal θ parameter (in black) in the initial (**A**) and iterative reweighting (**C**). The reweighted weights for each model from the MD ensemble (from the initial (**B**) and iterative (**D**) reweighting) plotted for different θ values, in black weights for optimal θ. (**E**) Root means square calculations calculated for the backbone atoms before and after the initial and iterative reweighting.

**
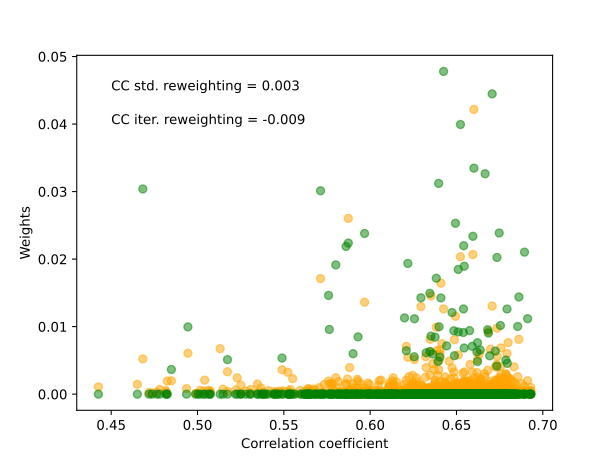
**

**Supplementary Figure 31**. Correlation between the weights of each model from the trigger factor MD ensemble upon the initial (orange) and iterative (green) reweighting and its level of agreement with the cryo-EM density expressed by correlation coefficient calculated in ChimeraX (CC).

**
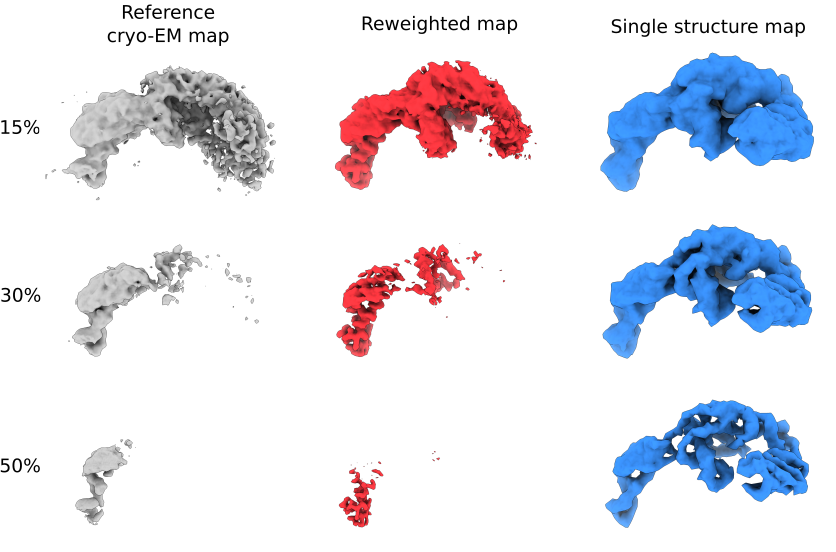
**

**Supplementary Figure 32**. The trigger factor reference cryo-EM map (in grey) presented along the calculated map for the posterior ensemble (in red) and single best-fit structure (in light blue) at different levels of the map threshold. Each map was normalised to enable comparison, and the selected threshold corresponded to the % of the maximum map value.


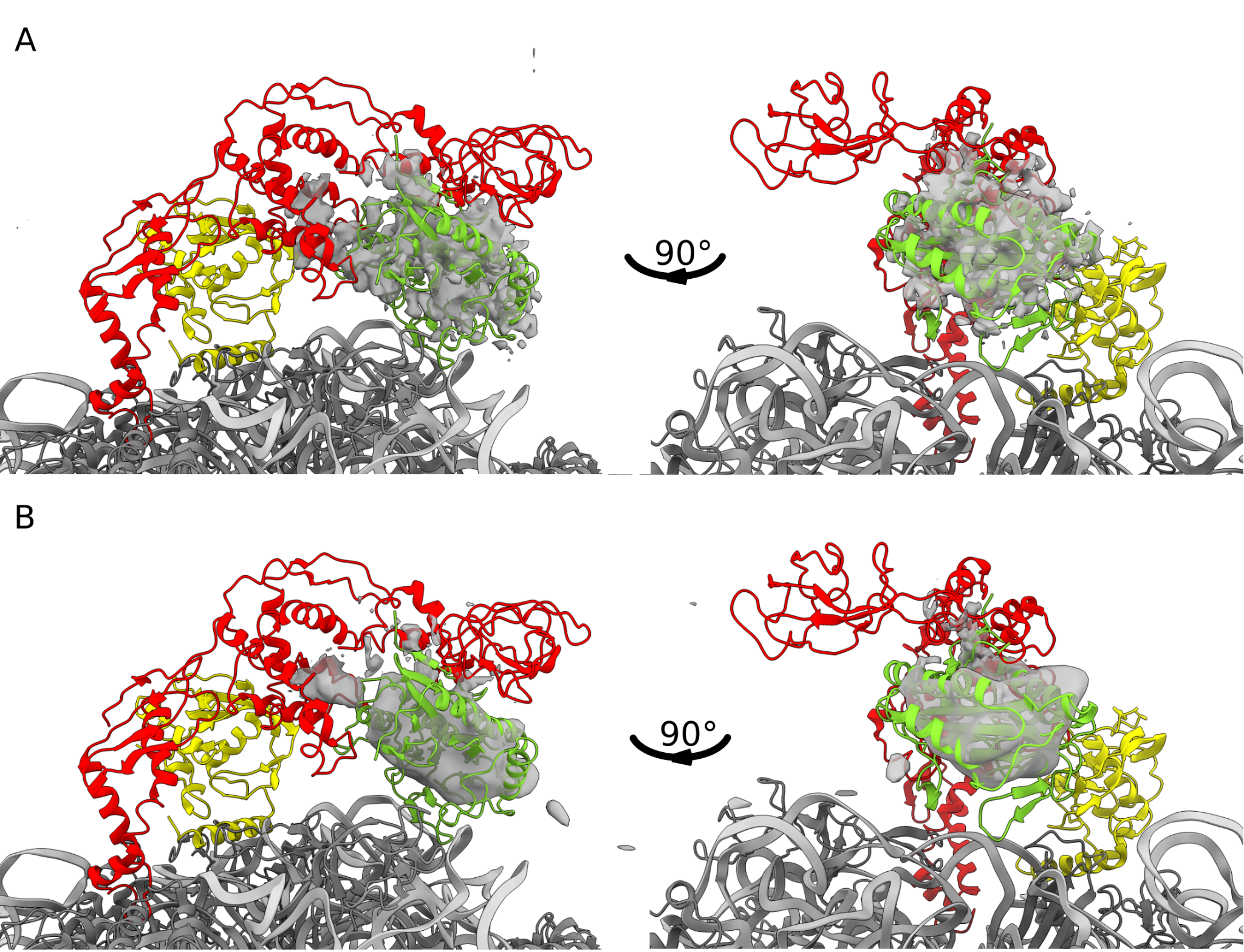


**Supplementary Figure 33**. X-ray MetAP structure (in green) fitted into the unaccounted cryo-EM density (in grey) from **(A)** EMDB:3061 and **(B)** EMDB:9778 maps. Additionally, we present 70S ribosome in grey (ribosomal proteins) and silver (RNA) alongside bound trigger factor in red and PDF in yellow.


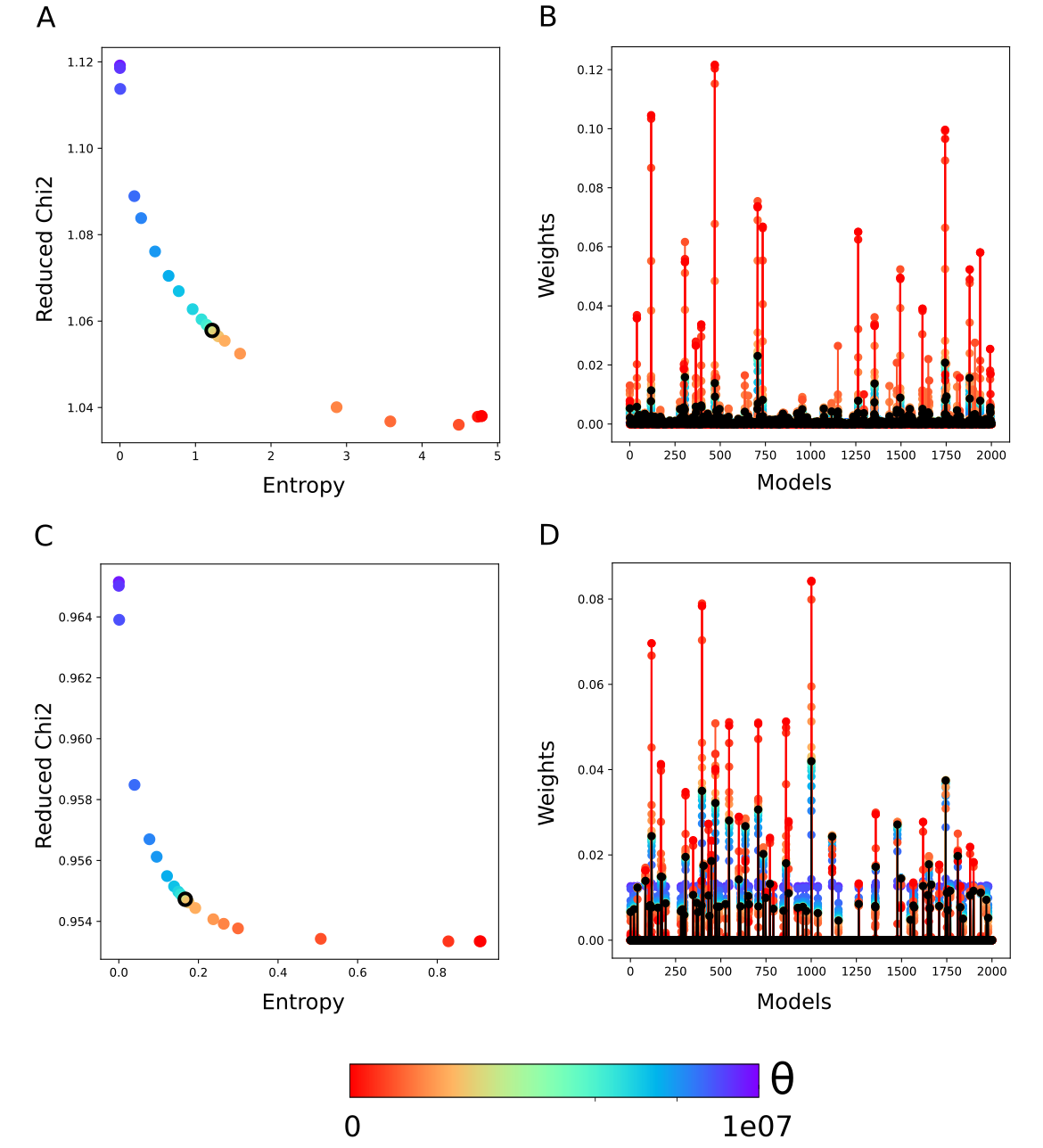


**Supplementary Figure 34.** Results of the TF+MetAP dataset reweighting. The L-curve analysis selects the optimal θ parameter (in black) in the initial (**A**) and iterative reweighting (**C**). The reweighted weights for each model from the MD ensemble (from the initial (**B**) and iterative (**D**) reweighting) plotted for different θ values, in black weights for optimal θ.


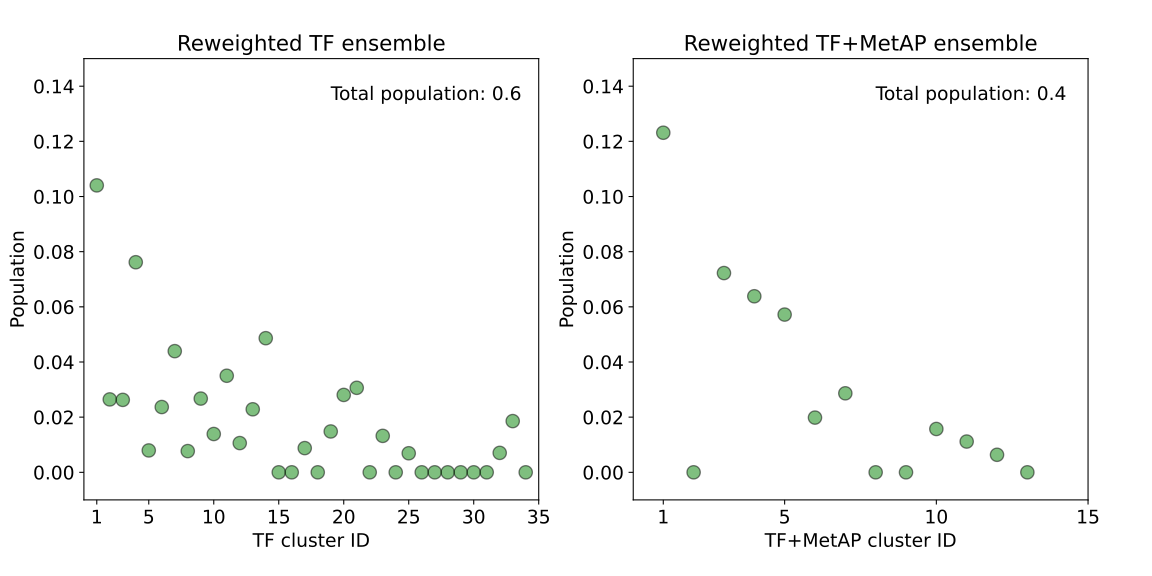


**Supplementary Figure 35.** Populations of the clusters of the TF and TF+MetAP reweighted ensembles.

**
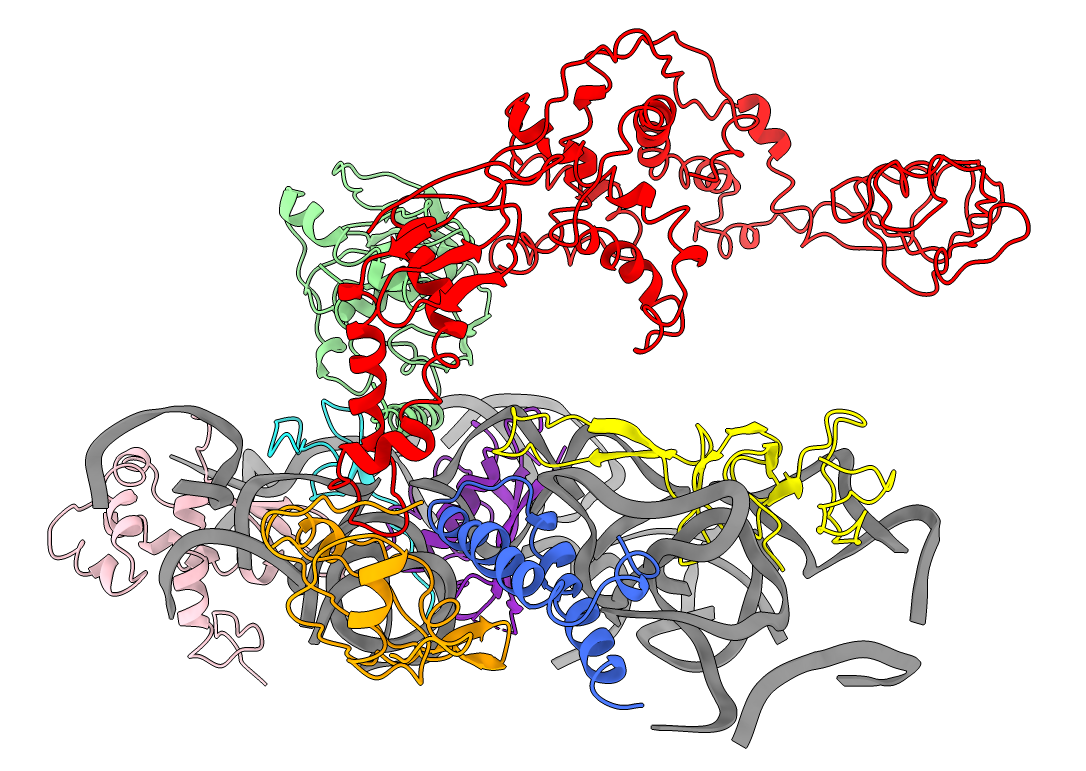
**

**Supplementary Figure 36.** The molecular system obtained from PDB ID: 7D80 for all-atom structure-based model MD simulation that encompasses part of the 70S ribosome surface including rRNA (in grey), uL24 (in yellow), uL29 (in light blue), uL23 (in orange), uL17 (in pink), uL32 (in cyan), uL22 (in violet) ribosomal proteins as well as trigger factor (in red) and peptide deformylase (in light green).

[**Bibliography**](https://sciwheel.com/work/bibliography)

[1. Müller, C. W., Schlauderer, G. J., Reinstein, J. & Schulz, G. E. Adenylate kinase motions during catalysis: an energetic counterweight balancing substrate binding. *Structure* **4**, 147–156 (1996).](https://sciwheel.com/work/bibliography/322311)

[2. Müller, C. W. & Schulz, G. E. Structure of the complex between adenylate kinase from Escherichia coli and the inhibitor Ap5A refined at 1.9 A resolution. A model for a catalytic transition state. *J. Mol. Biol.* **224**, 159–177 (1992).](https://sciwheel.com/work/bibliography/1560841)

[3. Ahn, M. *et al.* Modulating co-translational protein folding by rational design and ribosome engineering. *Nat. Commun.* **13**, 4243 (2022).](https://sciwheel.com/work/bibliography/13359631)

[4. Akbar, S., Bhakta, S. & Sengupta, J. Structural insights into the interplay of protein biogenesis factors with the 70S ribosome. *Structure* **29**, 755-767.e4 (2021).](https://sciwheel.com/work/bibliography/10778241)
